# Randomized control trial of *Tools of the Mind*: Marked benefits to kindergarten children and their teachers

**DOI:** 10.1371/journal.pone.0222447

**Published:** 2019-09-17

**Authors:** Adele Diamond, Chris Lee, Peter Senften, Andrea Lam, David Abbott

**Affiliations:** Program in Developmental Cognitive Neuroscience, Department of Psychiatry, University of British Columbia, Vancouver, Canada; Virginia Commonwealth University, UNITED STATES

## Abstract

The kindergarten program, *Tools of the Mind* (*Tools*), has been shown to improve executive functions (as assessed by laboratory measures) and academic performance. The objective here was to see if *Tools* can improve executive functions in the real world (in the classroom), academic outcomes not previously investigated, reduce bullying and peer ostracism, and increase teachers’ and students’ joy in being in the classroom. This first randomized controlled trial of *Tools* in Canada included 351 kindergarten children (mean age 5.2 years at entry; 51% female) in 18 public schools. Stratified randomization resulted in teachers and students in both groups being closely matched. Teachers in both groups received the same number of training hours and same funds for new materials. Outcome measures were pre and post standardized academic skill assessments and teacher online survey responses. This study replicated that *Tools* improves reading and shows for the first time that it improves writing (far exceeding levels the school districts had seen before), self-control and attention-regulation in the real world (e.g., time on task without supervision), reduces teacher burnout and children being ostracized or excluded, and increases the joy students and teachers experience in school. By Spring, *Tools* teachers were still enthusiastic about teaching; control teachers were exhausted. These results were not only better than the control group but also better than *Tools* teachers experienced the year before *Tools*. Thus, children in a kindergarten curriculum that emphasized play, improving self-regulation, working together and helping one another, and hands-on learning performed better academically, showed less bullying and peer ostracism and more kindness and helping behavior than students in more traditional classes, and teacher enthusiasm for teaching soared. *Tools* reduced initial disparities separating children, schools, and teachers.

## Introduction

Self-control and attention-regulation in early childhood are highly predictive of school performance [1–4], workplace success [5, 6], health [6–8], and life satisfaction [9–11]. They are often more predictive than IQ [6, 12, 13] or socio-economic status (SES) [6, 14]. Children who enter school with poorer academic skills and poorer self-control and attention-regulation quickly fall behind and the gap progressively increases in school achievement [14, 15] and health [16, 17]. Hence there is great interest in helping children enter Grade 1 with the academic and executive function (EF) skills they need to launch them on a positive trajectory.

Similarly, social-emotional well-being in childhood predicts better school performance [18–21] and better outcomes on diverse variables in adults [19]. Student bullying and peer exclusion are major social and mental health concerns [22, 23] and classroom stress is causing teachers to burn-out and leave the profession in unprecedented numbers [24–26]. Hence there is great interest in improving students’ camaraderie and kindness, and reducing stress, in the classroom.

Much of the focus has been on prekindergarten programs [27–32]. Kindergarten represents a much less-studied context for investigating ways to improve social-emotional and EF competencies. Yet free, public kindergarten is available throughout most developed countries. At least one longitudinal study reports that attending a higher quality kindergarten is associated with higher rates of college attendance and higher earnings in adulthood [33].

This paper reports the results of a study that investigated whether the *Tools of the Mind* (*Tools*) kindergarten program could improve self-control and attention-regulation, academic performance, prosocial behavior, and reduce classroom stress and teacher burnout.

Reducing stress and increasing social harmony are not only important as factors that improve EFs and academic performance, but are important goals in their own right. A program that can reduce ostracism and bullying and reduce teacher burnout is one worth taking a look at. Such a program was examined here.

To unpack the terms used above a bit, self-control and attentional control comprise the “inhibitory control” component of EFs [34]. Self-control involves resisting temptations (including all the temptations not to stay on task or see it through to completion) and resisting speaking or acting reflexively (e.g., instead of responding immediately, giving oneself time to think or calm down before acting). Attentional control involves resisting distractions, being able to pay attention and stay focused for an extended period.

These inhibitory control abilities are critical for success in school [1–4] and in social relations [35–37]. They are needed for inhibiting all the pulls not to pay attention or stay focused and also for complying with school norms, such as staying seated or raising one’s hand, and social norms, such as not grabbing what someone else has or not talking while someone else is speaking. This is probably one of the many reasons why EFs, social-emotional competence, and academic performance are highly interrelated, e.g. [38–40].

The other EFs are working memory, cognitive flexibility, reasoning, and planning [34], but it is inhibitory control that is most predictive of long-term outcomes [6, 7]. A reasonable prediction is that a school program that improves inhibitory control, in addition to addressing academic skills, should produce better academic outcomes than programs that address academic skills but do not address inhibitory control or do so less successfully. We tested that prediction here.

If a person feels lonely or rejected, or is stressed or sad, that negatively impacts inhibitory control, academic performance, and physical and mental health (evidence that loneliness impairs EFs and specifically inhibitory control [41–43], academic performance [20, 44], and health [42, 45–47]; evidence that stress impairs EFs and specifically inhibitory control [48–50], academic performance [51–53], and health [54–56]; evidence that prolonged sadness impairs EFs and specifically inhibitory control [57–59], academic performance [60, 61], and health [62–65]. Therefore, a reasonable prediction is that in a school program that promotes students working together and being kind to and supporting one another (i.e., prosocial behavior [66]) one should find less peer rejection, more joy in the classroom, less teacher burnout, and better student academic performance and inhibitory control.

*Tools* is a kindergarten curriculum that focuses as much on improving EFs (especially inhibitory control), classroom climate, prosocial behavior and interpersonal skills as on improving academic skills. There is already evidence that it improves EFs, academic performance, and teacher-child relationships and reduces aggression [67–69], though when only parts of the program have been implemented as an add-on to the curriculum, those benefits have not been observed [70, 71].

Three independent evaluations of *Tools* have been published. The first, published in *Science* [69], found that recent graduates of *Tools* showed much better attention-regulation on a Flanker-type task (85% vs. 50% correct) than controls. Children were not evaluated before the intervention so it is possible that children in *Tools* had better attention-regulation at the outset, though the groups were closely matched on many demographic variables. At-risk, low-income children had been randomized to *Tools* or to another new curriculum that the school district had developed and predicted would outperform *Tools*. One school became so impressed by how much *Tools* children were out-performing others that they dropped out of the study and switched all kindergarten classes to *Tools*, feeling it unethical to deprive any of *Tools*.

A much larger study [67, 68] found better and more improved vocabulary, math, teacher-reported teacher-child relationships, and emotion-regulation on the dot-probe task. They did not find, however, better or more improved inhibitory control or cognitive flexibility on the Hearts and Flowers task, card sorting, or Flanker tasks in kindergarten children in *Tools* versus controls. They also found less and more reduced teacher-reported conduct problems or aggression in kindergarten children in *Tools* versus controls. Academic benefits were even larger the following year (Grade 1), where gains in reading first became evident. Effects were about eight times larger in low-income schools.

The third study [72] compared a daycare-based *Tools* program for children 3–4 years old to a high-quality, existing play-based program. Children in *Tools* whose parents rated them as highly hyperactive and/or inattentive in the Fall showed greater gains on an inhibitory control task of self-control than control children. The authors concluded that “*Tools* may be advantageous in classrooms with children experiencing greater challenges with self-regulation, at no apparent cost to those less challenged in this regard” (p. 2).

We predicted we would find benefits from *Tools* on important variables not previously investigated: (a) the academic skill of writing, (b) camaraderie and helping one another in the classroom, or its flip side reduced peer ostracism and exclusion, (c) teachers’ joy in teaching, (d) students’ joy in learning, and (e) EFs in the real-world versus on laboratory measures (specifically the ability to inhibit distraction in the classroom and stay on task), in addition to replicating previously demonstrated benefits to reading. We predicted that classrooms with less play, hands-on learning, or incorporation of training and scaffolding of EFs in school activities, even if they spent more time on academic content, would be less successful in improving academic outcomes and would be characterized by greater stress in the classroom and more teacher burnout.

### Research design

The year before implementation, all public elementary schools in Vancouver and Surrey, the two largest school districts in British Columbia (BC), Canada, were queried to see if a kindergarten teacher at the school was interested in implementing *Tools* and if the principal was also supportive of that. All schools where both the principal and at least one kindergarten teacher responded ‘yes’ were included in the pool from which random selection was made. This was done because one would expect implementation of *Tools* to be poor where the teacher or principal did not want it, and the strong teachers’ union would not allow teachers to be told to implement a test curriculum they did not want.

Because teachers, principals, or schools open to implementing *Tools* might differ from those unwilling to go to the effort to learn and implement a new curriculum, we also selected the control schools from the same pool of schools. Within each city, pairs of closely-matched schools were created from this pool (matched on the relevant kindergarten teacher’s years of experience and training and on socio-economic characteristics of kindergarten children at the school including ethnicity, subsidized lunch status, and home language). Ten pairs were randomly selected and one member of each was randomly assigned to implement *Tools*.

This study had human subjects research ethics approval from the University of British Columbia, Vancouver School Board, and Surrey School Board. Informed written consent was obtained from all teachers for their participation and all principals for their school’s participation. The only data from children were their scores on BC assessment tools and their ESL and subsidized-lunch status, which the school districts collects as part of their educational mission, and which we received aggregated by classroom. Since we did not collect any data directly from the children we did not request consent from them or their parents.

One pair dropped out a couple of months into the school year. Both teachers had personal, family reasons for not being able to participate in the study. We thought it would be too difficult for a teacher new to *Tools* to catch up at that point, so did not replace that pair.

### Control condition: Existing curriculum in BC kindergartens + special workshops

Kindergartens in BC are all full-day. Most kindergartens have 20–22 students. All follow the same prescribed learning outcomes and principles of appropriate practice [73]. Thus, the curriculum is the same in Vancouver and Surrey, and in *Tools* and control classes. The BC Ministry of Education is committed to educating children not just in academics but also in social responsibility. Most teachers (89% of control teachers and 77% of *Tools* teachers) had received training in the *Second Step*^®^ social-emotional learning (SEL) program that teaches social skills, empathy, and emotion management [74]. Additionally, 56% of control teachers and 50% of *Tools* teachers had received training in the *MindUp*^™^ program (which teaches social and emotional skills and mindful awareness) [75].

There was play in control classes, but it was usually unsupervised or scripted, not as in *Tools*. (For example, a child in *Tools* might record a plan to play an astronaut today. Early in the year, he might abandon that after 1–2 minutes to play something else. In control classes that would be fine. In *Tools*, the teacher comes over with the child’s plan, “You need to follow through with your plan. You can be something else tomorrow.” Children in control kindergartens do not tend to make plans. By the Spring, *Tools* children sustain make-believe dramatic play for 25–30 min without adult guidance; control children tend to do so for only a few minutes).

Control kindergartens had more ‘whole group’ activities. In *Tools* kindergartens, children worked more independently in pairs or small groups. Control kindergartens used rewards (e.g., gold stars); *Tools* does not. Time-outs are used in control classes, but not in *Tools*.

### Experimental condition: *Tools of the Mind*

The *Tools* curriculum, which exists only for preschool and kindergarten, is grounded in the idea that social-emotional development and improving EFs, especially inhibitory control, is as important as teaching academic skills and content. Developed by educational psychologists, Bodrova and Leong [76], *Tools* is based on the work of Vygotsky [77, 78] and has been revised and improved over 23 years of iterative research and implementation.

Vygotsky emphasized that cognitive and social development are fundamentally intertwined and that social interactions are key to developing EFs and cognitive skills, thus in *Tools* there are not separate activities for academics and SEL, rather activities address both. That makes *Tools* rather unique. Tools teachers are taught how to foster paired activities and an atmosphere of cooperation and mutual support. A major difference between *Tools* and traditional kindergarten is the far greater use of peer social interaction for learning in *Tools*–two children helping one another, cooperating in learning the material together or in one teaching or checking the other. Children learn to help bootstrap one another’s EFs, providing helpful reminders to each other. Consistent with Vygotsky’s view that language is central to EF development, Tools provides specially designed opportunities for children to talk to each other, thus aiding the development of oral language as a tool for social interaction and encouraging the emergence of private or “inner” speech that serves as a mechanism for self-regulation [79, 80].

Vygotsky also emphasized the importance of social pretend play (e.g., playing doctor and patient or grocery store) for the development of EFs in young children. It is an important component of *Tools*. The quantity and quality of social pretend play in *Tools* distinguishes it sharply from traditional kindergarten. Children enact roles with implicit rules, role speech, and the use of symbolic props (e.g., a block might be a phone or a loaf of bread). Mature make-believe play challenges and helps build all three core EFs: Children must inhibit acting out of character (inhibitory control), hold in mind the role they’ve chosen and those of others (working memory), and flexibly adjust as their friends take the scenario in unexpected directions (cognitive flexibility).

Each child is paired with every other at least once every week in *Tools*. Students adapt to the personal quirks of their classmates. They know if they are not paired with their favorite person it won’t last long, everyone will also be paired with this person, and soon they will be paired with someone else, so complaining about being “stuck” with someone (so common in the early grades) is absent.

Another marked difference between *Tools* and traditional kindergarten is the far greater time children spend in hands-on learning and far less time in teacher-led whole-group activities in *Tools*. As one teacher put it, with *Tools* she is the “Guide on the Side” rather than the “Sage on the Stage.” At any age we learn something better when we need it for what we are doing [81, 82]. For young children that is particularly important because they have such difficulty sitting and listening for any length of time.

Because children can work on their own or with one or two others, teachers can provide individualized instruction and assessment. A *Tools* teacher helping one child is not taking time away from others because others are engaged in meaningful activity. Because children can work on their own they can proceed at their own pace, without rushing other children or holding them back. The use of self-correcting materials enables children (or their “study buddy”) to detect and correct errors without the teacher having to tell them.

Rather just assessing a child’s current level of competence (as do standard assessments), *Tools* teachers use dynamic assessment to determine a child’s readiness to advance or why the child is having difficulty grasping something. This consists of a series of prompts and hints to probe children’s skills and understandings that are “on the edge of emerging [78].”

Weekly one-on-one Learning Conferences with the *Tools* teacher engage the child in planning his/her own education, empowering the child to take a lead role. Children “talk through” both correct and incorrect answers, helping them learn to reflect on and correct mistakes. In these conferences errors are treated as valuable learning opportunities, not anything to be embarrassed about.

A distinguishing feature of *Tools* is the absence of extrinsic incentives, such as stickers or gold stars. The *Tools’* philosophy is that learning and developing mastery are intrinsically rewarding, and that external rewards would convey the wrong message.

An example of paired peer-social interaction in learning activities as well as how training EFs is seamlessly incorporated to *Tools* academic activities is the *Tools* literacy activity called “Buddy Reading.” Children pair up to take turns “reading” their picture book to one another. With each child eager to tell his or her story; no one wants to listen. To help the children succeed at exercising inhibitory control, the teacher provides scaffolds (one child per pair gets a line drawing of lips and the other a drawing of an ear); the teacher explains that “ears don’t talk; they listen.” This enables the child with the ear to inhibit talking and to listen. Children then trade drawings and roles, thus learning to enact the social norms of taking turns and waiting one’s turn. After a few months, the pictures are no longer needed; children can succeed without them.

This illustrates another key aspect of *Tools*: Rather than letting children flounder, teachers provide supports (scaffolds) so that most children, regardless of ability level, succeed. Concrete visual signs and symbols help bootstrap fragile working memory and language skills. Classroom materials have few distractions, thus making attention regulation easier. These supports are gradually removed as children improve. Thus children succeed, instead of experiencing failure or criticism. The boost to self-confidence and self-esteem from experiencing success is one key element of *Tools*. Indeed, testers in one study of *Tools* [69] could tell which children had been in *Tools* because on the most difficult conditions control children gave up but *Tools* children insisted, “I know I can do this. Let me try again.”

Because scaffolds and other children help students inhibit their impulsive behaviors and act appropriately, *Tools* teachers have less worries about students misbehaving; they can relax. Having fewer worries about being reprimanded, the children can relax.

For those wanting more information, S1 File provides a brochure about *Tools*.

### Comparability

We went to lengths to treat both *Tools* and control teachers comparably. *Tools* teachers received a three-day workshop on *Tools* before the school year began. We offered control teachers three days of workshops at the same time on whatever they wanted. They made suggestions and voted on them. Their workshops received excellent reviews from the teachers. (They chose one-day workshops on “Using Technology in your Kindergarten Classroom,” “Teaching Children with Autism Spectrum Disorder,” and “NOT your typical approach to Math in Kindergarten.”) Both groups of teachers were comparably compensated for their time in attending the three days of workshops. The four one-day workshops for *Tools* teachers during the school year were held on Professional Development Days when school districts arranged for instruction and enrichment programs for teachers.

Kindergarten classes in the US usually have a teaching assistant besides the teacher; kindergartens in BC do not. *Tools* needs such an assistant for the 90-minute literacy block each morning. Therefore, we paid a token $30/day for kindergartens in both groups to have an assistant for 90 minutes daily. Typically the assistant was a relative of one of the children in the class or a friend or relative of the teacher. Teachers in the *Tools* group needed to purchase supplies. Therefore, all teachers in both groups received an allowance of $1,000 to purchase supplies for their classroom. All funds for this came from the BC Ministry of Health and BC Mental Health Foundation.

There was one unintended difference between the *Tools* and control groups: *Tools* teachers chose on their own to meet together a few times during the school year (besides when there was a workshop)—thus providing social support and enabling each to learn from one another. This probably helped less-experienced teachers to do so well with *Tools*. (Had we known about these meetings, we would have arranged for similar meetings for control-group teachers).

### Assessments

Pre-intervention levels of the children on language and math skills and on behavioral control and sociability were determined within the first month of school. Post-intervention levels were determined eight months later (May 5–15). Academic skills were assessed using BC’s objective, standardized assessment tools [83] including the Developmental Reading Assessment (DRA2)^™^ [84] (see S2 File). These results were also obtained for the pre-*Tools* year for the classes taught by teachers assigned to *Tools*. Reading and writing were done in English. Students’ attitudes and behavior were reported by teachers. Teachers responded to an online survey (using the Survey Monkey platform) with multiple-choice questions and open-ended opportunities to elaborate. The survey questions are provided in S3 File.

### Data analyses

Since randomization was at the level of schools, analyses of student outcomes were nested within schools. Since the data were often ordinal, binary, or not normally distributed, in most cases the generalized estimating equation was used for data analyses, as it provides valid inferences regardless of the data distribution and is robust for both parametric and non-parametric analyses. Chi-squares were generated from the generalized estimating equation within a poisson loglinear model when the data distribution was skewed, or, for categorical data, a binary logistic model. For interval data, where the data were roughly normally distributed and the variances roughly equal between groups or could be made so by a transformation such as arcsine, analysis of variance (ANOVA) was used to compare one group to other. Linear regression was used for the analysis of whether *Tools* helped the children more behind in reading more than those who started out reading at a higher level.

S4 File presents the results for all of our statistical analyses controlling one at a time for free-lunch status, ESL status, and years of teaching experience. With nine classrooms per condition, we do not have the power to control for more than one covariate at a time. Free-lunch status was occasionally related to our outcome measures, as was ESL status, years of teaching rarely. All analyses are reported in this paper controlling for free-lunch status (as a proxy for lower SES). To see the results controlling for ESL status or years of teaching please refer to S4 File.

Since the dependent measures are interdependent and interrelated, one could argue that correcting for multiple comparisons is not needed. On the other hand, with several dependent variables we felt some correction should be applied. As a compromise between those two viewpoints, we have divided the normal significance level in half and required p < 0.025 for a result to be considered statistically significant. To help illuminate the reasons behind why statistical differences were found and to put a human face on them, direct quotations from teachers’ survey responses are included in S5 File.

## Results

### Descriptive statistics

Teachers and students were well matched in the two groups. See Table 1. Most teachers in both groups were outstanding and very experienced. There were nine teachers (schools) per group; 172 children in the *Tools* group; 180 children in the control group.

**Table 1:** Descriptive statistics.

Measure	Tools teachers	Control-group teachers
Means years of teaching (SD)^A^	16 (4.9)	15 (7.4)
Range of years of teaching	1–20	7–29
Mean years of teaching kindergarten (SD)	7 (3.6)	8 (4.1)
Range of years of teaching kindergarten	1–15 years	2–13 years
Mean # of children in each class (SD)	19 (2.0)	20 (1.4)
Range of # of children in each class	17–22	18–22
Total number of children in each group	172	180
Percentage of girls in each group	50%	52%
Mean age in years of kindergarten students on Sept. 15 (SD)	5.03 (0.5)	5.10 (0.6)
Mean # of special-needs children per class (SD)	2.0 (1.0)	2.5 (1.9)
Range of # of special-needs children per class	0–4	0–5
Mean # of ESL^B^ children per class (SD)^C^	6.5 (5.5)	13.0 (5.7)
Range of # of ESL children per class	0–14	2–19
Mean # children on subsidized lunch (lower income)/class (SD)^D^	4.2 (1.6)	1.8 (2.0)
Range of # of children on subsidized lunch per class	0–12	0–10
# of classes with no child on subsidized lunch	6	8
# of classes with 44% of children on subsidized lunch	1	0
# of classes with 53–55% of children on subsidized lunch	1	1
# of classes with 71% of children on subsidized lunch	1	0

^A^ SD = standard deviation

^B^ ESL = English as a second language

^C^ There were more ESL children in the control group than in the *Tools* group (F[1,16] = 6.52, p < 0.02, partial eta squared [ηp^2^] = 0.31).

^D^ There was a tendency for more lower-income children to be in *Tools* classes than control classes (F(1,16) = 4.46, p < 0.05, ηp^2^ = 0.25).

### Reading

At the beginning of kindergarten, most children could not read even the simplest words. Most classes had no child who could read more than the simplest sentences; the exceptions were one *Tools* class and three control classes which each had three children who could read at a higher level. No significant difference in reading skills was found between *Tools* and control classes in September.

**Fig 1:**
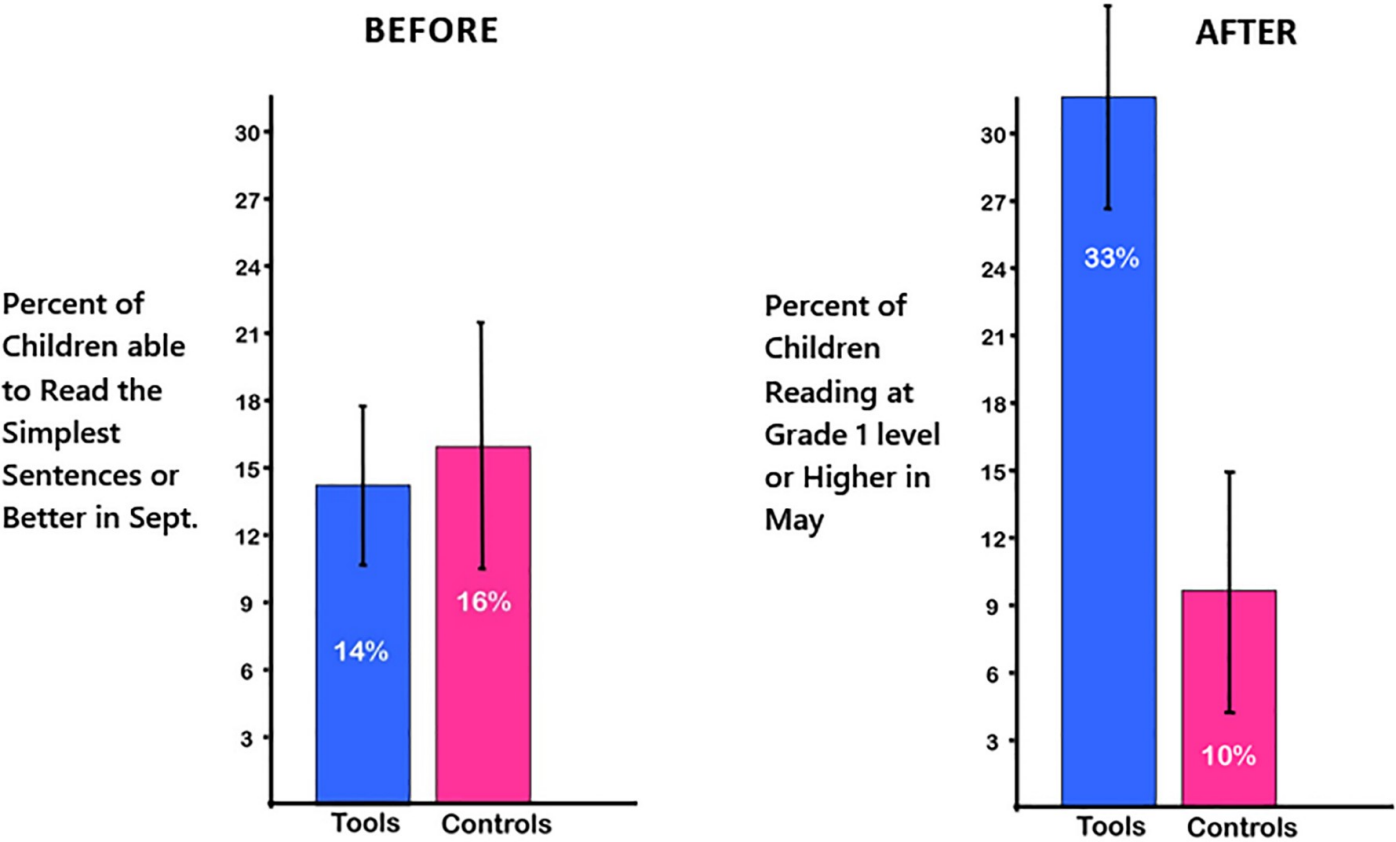
Reading skills. By May, three times as many children in *Tools* than in control classes were reading at Grade 1 level or better, although both groups started out comparably in the Fall.

By May, eight of the nine *Tools* classes had more than two children reading at Grade 1 level or higher, while only one of the nine control classes had more than two children reading at Grade 1 level or higher. Children in *Tools* made significantly greater progress in reading than children in the control group (χ^2^(1, N = 18) = 4.64, p = 0.02, odds ratio = 3.25). Three times more children were reading at Grade 1 level or higher by May in *Tools* classes than in control classes (33% vs. 10%): F(1,15) = 6.67, p < 0.02, partial eta squared (ηp^2^) = 0.33. See Fig 1. Conversely, almost three times more children were still non-readers by May in control classes than in *Tools* classes (28% vs. 10%): F(1,15) = 6.02, p = 0.02, ηp^2^ = 0.29.

This better progress in reading with *Tools* was also reflected in comments by teachers and parents (see S5 File–Comments by Teachers, Parents, and Principals). Most *Tools* teachers said they had never seen progress like this in reading before.: “The literacy level in the classroom this year is much higher [than in past years].”

Lower-income children in *Tools* (those receiving subsidized lunch at school) did not show greater progress in reading than did children in *Tools* from more prosperous homes (χ^2^(1, N = 9) = 4.17, p = 0.12 [NS], odds ratio = 2.05; here the covariate was ESL status instead of subsidized lunch). With only nine Tools classes, though, there was limited power to detect a difference. The reading of those who started farther behind in September, however, showed far more progress than the reading of those who started out reading at a more advanced level, as the regression of the difference in reading level (May minus September) against reading level in September shows: F(2,6) = 18.18, p < 0.005, R^2^ = 0.89.

### Writing

**Fig 2:**
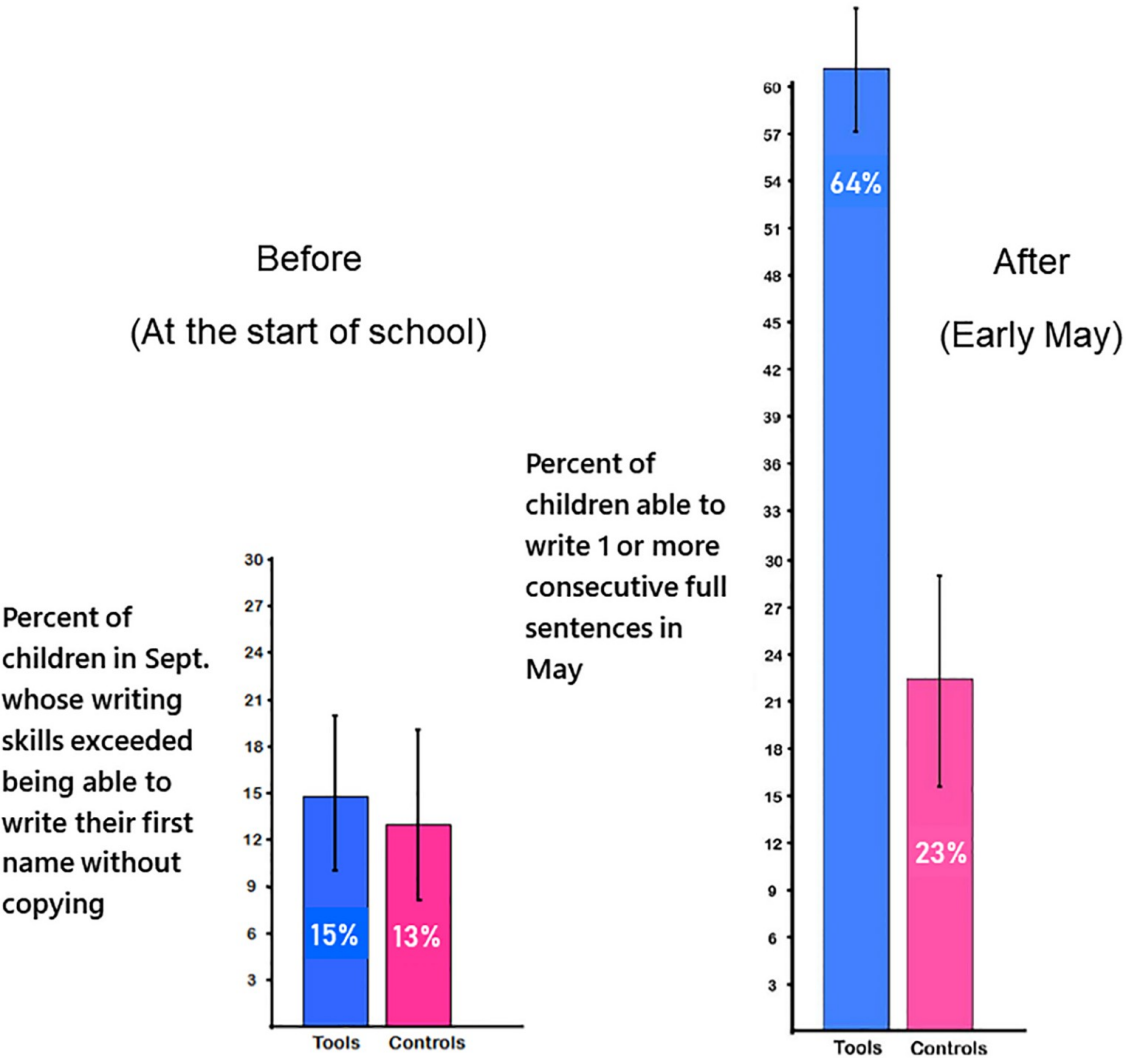
Writing skills. By May, three times more children in *Tools* than in control classes were able to write a full sentence they themselves composed or multiple consecutive ones (F[1,15] = 18.10, p < 0.001, ηp^2^ = 0.55).

Children in the two groups started out similarly in writing ability (χ^2^(1, N = 18) = 1.45, p > 0.20 [NS], odds ratio = 1.10). In September, roughly three children per class in both groups (range = 1–5) could do no better than scribble. In 67% of *Tools* classes and 56% of control classes, most children could write their first name without copying (85% of children in *Tools* and 87% of control children). By May, almost all children in both groups could do better than that. The difference was in how far they had progressed. Children in Tools progressed much farther (χ^2^(1, N = 18) = 20.20, p < 0.001, odds ratio = 26.18). Three times as many children in *Tools* versus control classes reached as far as being able to write a full sentence they themselves composed with most sounds represented (30% vs. 10%). Almost three times as many children in *Tools* versus control classes progressed further than that; they could write 2 or more consecutive sentences they composed with most sounds represented (33% vs. 12%). More children in *Tools* than in control classes progressed from September to May to being able to write a full sentence or multiple consecutive ones that themselves composed with most sounds represented: F(1,15) = 18.10, p < 0.005, ηp^2^ = 0.55. See Fig 2.

**Fig 3:**
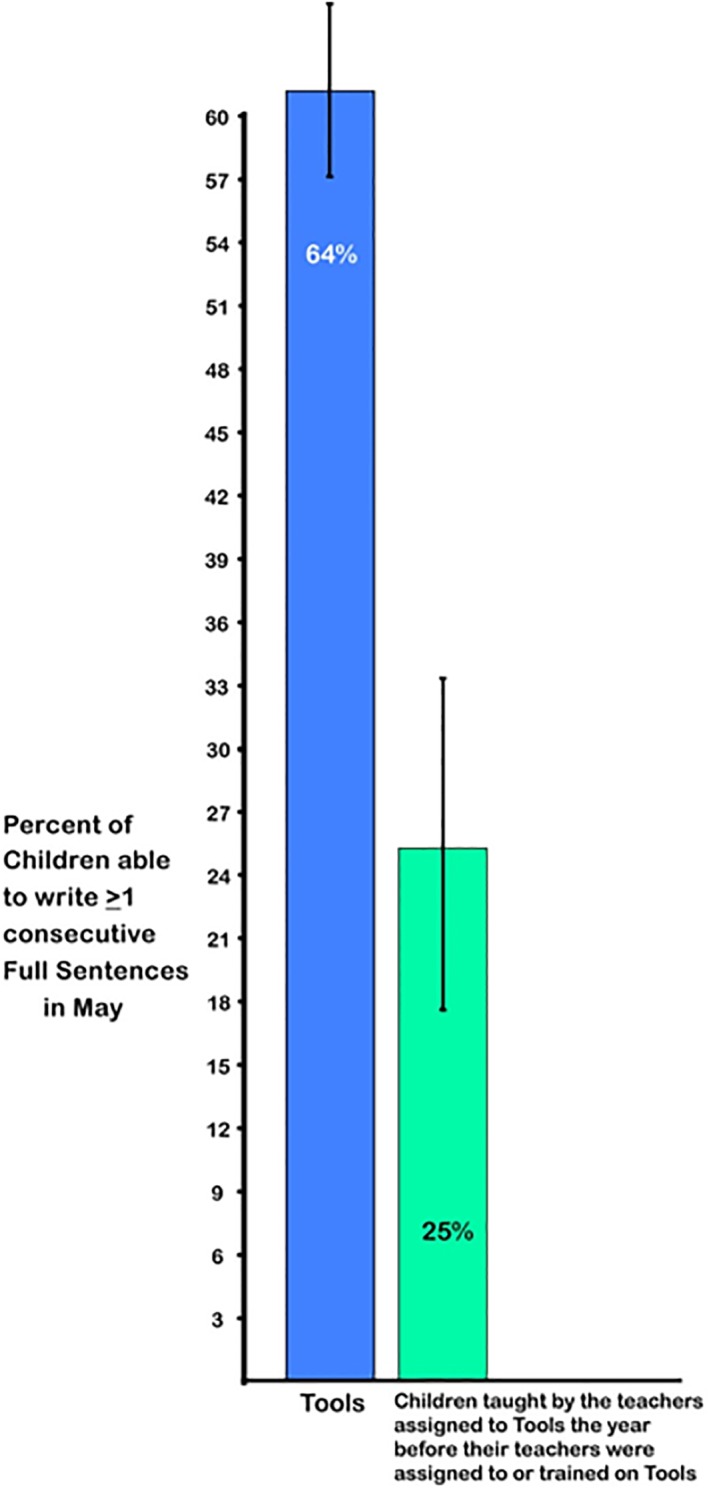
Percentage of children able to write at least one sentence they composed, comparing outcomes for children taught by the teachers assigned to *Tools* in the pre-*Tools* year (before *Tools* was implemented) and Year 1 of *Tools*. Children taught by the same teachers the year before those teachers implemented *Tools* were significantly less advanced in writing than children taught by those teachers the next year when they were implementing *Tools*: χ^2^(1, N = 8) = 13.54, p < 0.01, odds ratio = 9.42).

It is not so surprising that the writing of children in *Tools* advanced further than the writing of control children since *Tools* emphasized writing and control classes did not, though the advanced level of writing by children in *Tools* would astonish most kindergarten teachers. Indeed, we had to add questions about children’s writing skills to the online teacher survey because the writing levels achieved by children in *Tools* exceeded the upper limits on the BC assessment tools for kindergarten. Teachers reported never having seen writing progress like this before (see S5 File) and the data bear them out (see Fig 3).

### Math

Both groups started out with virtually no math skills. Because of the complexity of implementing *Tools* for the first time in Canadian kindergartens, and because of a decision to concentrate on language skills, math was not a focus of the *Tools* program in Year 1 of its implementation in BC. Thus, the developers of *Tools* and local *Tools* coaches did not expect *Tools* children to advance more in math than did controls, but they came close to doing so: In eight of the nine *Tools* class, most children progressed to being able to do simple addition and in four of the nine most progressed even farther to simple subtraction. In no control class were most children able to do simple subtraction. At the lower end of continuum, in only two *Tools* class were most children able to do no better than to count up to 20 objects, while that was true for four of the nine control classes. Neither the difference between Tools and control classes in the percentage of children who could do no better than count up to 20 objects in May nor the difference in the percentage who could do simple subtraction by May reached significance however (F(1,15) = 3.16, NS; F[1,15] = 1.17, NS; F[1,15] = 1.77, NS, respectively).

### Social inclusion and other prosocial behavior

Both groups started off comparably. *Tools* teachers reported that in the Fall they had 3–8 children who had difficulty interacting in the classroom (mean (SD) = 5 (0.7) per class; 26%). Control teachers reported that they had 0–9 children who had difficulty interacting in the classroom (mean (SD) = 4 (2.5) per class; 20%). By May, the percentage of children reported to be having problems interacting was lower in Tools than in control classes (F[1,15] = 6.83, p < 0.02, ηp^2^ = 0.31) and it had gone down much more in *Tools* than in control classes (F[1,15] = 20.59, p < 0.001, ηp^2^ = 0.58).

Tools teachers commented, for example: “In years past, they have not helped each other to this degree. This year I have witnessed many students going to another student’s aid.” “They offer help and assistance when needed without being asked and without belittling the struggling student. They look out for one another and ensure everyone has someone to play with or talk to.” “They are cheering each other’s success, are more supportive of each other.” “More of a sense of community [this year]. I see children helping each other and looking after each other to a greater degree from in the classroom to out on the playground at recess [than in past years].” See more comments on this topic in S5 File.

On the other hand, control teachers commented, for example: “[We] have a few children who have a very difficult time acting kind most of the time. This makes it difficult to have a totally close knit community, as these children, while they have progressed, still need significant support to make choices that benefit everyone and not just themselves.” “The students are learning to read and write, but their ability to be well-adjusted and considerate human beings lags behind.” More comments by control teachers are provided in S5 File.

**Fig 4:**
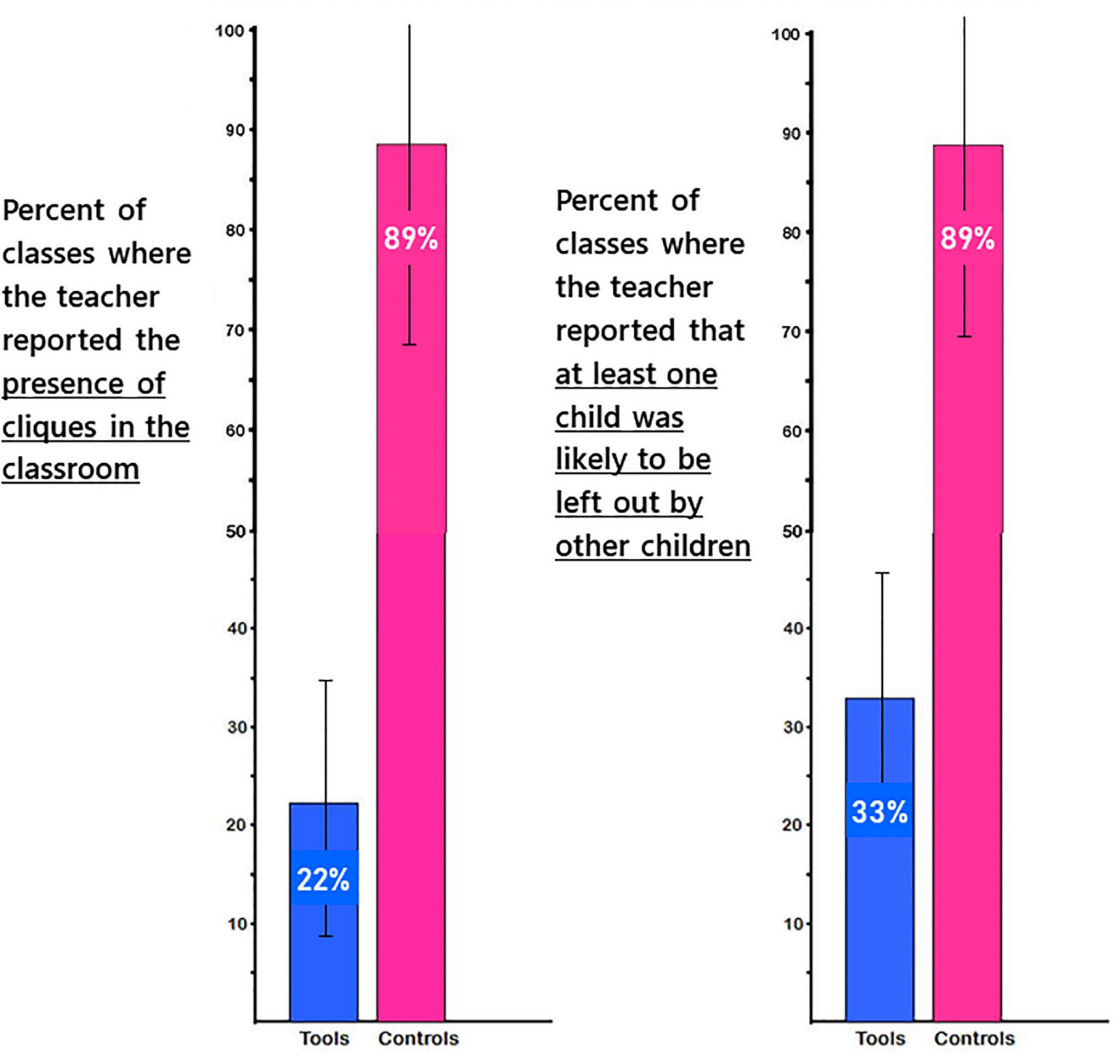
Teacher reports of peer rejection and presence of cliques about here. In-groups and out-groups and peer rejection or exclusion were the norm in control classes and rare in *Tools* classes.

Only 22% of *Tools* teachers reported the presence of cliques in their classes compared with 89% of control teachers. The difference in the incidence of teacher-reported cliques was significant: χ^2^(1, N = 18) = 11.99, p < 0.001, odds ratio = 15.77). Fully 89% of control teachers reported in May that there was at least one child in their class who tended to be ostracized or left out; only 33% of *Tools* teachers reported that. Instances of a student being left out or ostracized were noticeably more common in control *versus* Tools classrooms: (χ2(1, N = 18) = 4.87, p = 0.02, odds ratio = 3.45). Teachers’ comments echo the stark difference evident in Fig 4 (see S5 File).

### Attention-regulation and self-control

#### Ability to get back to work after a break

**Fig 5:**
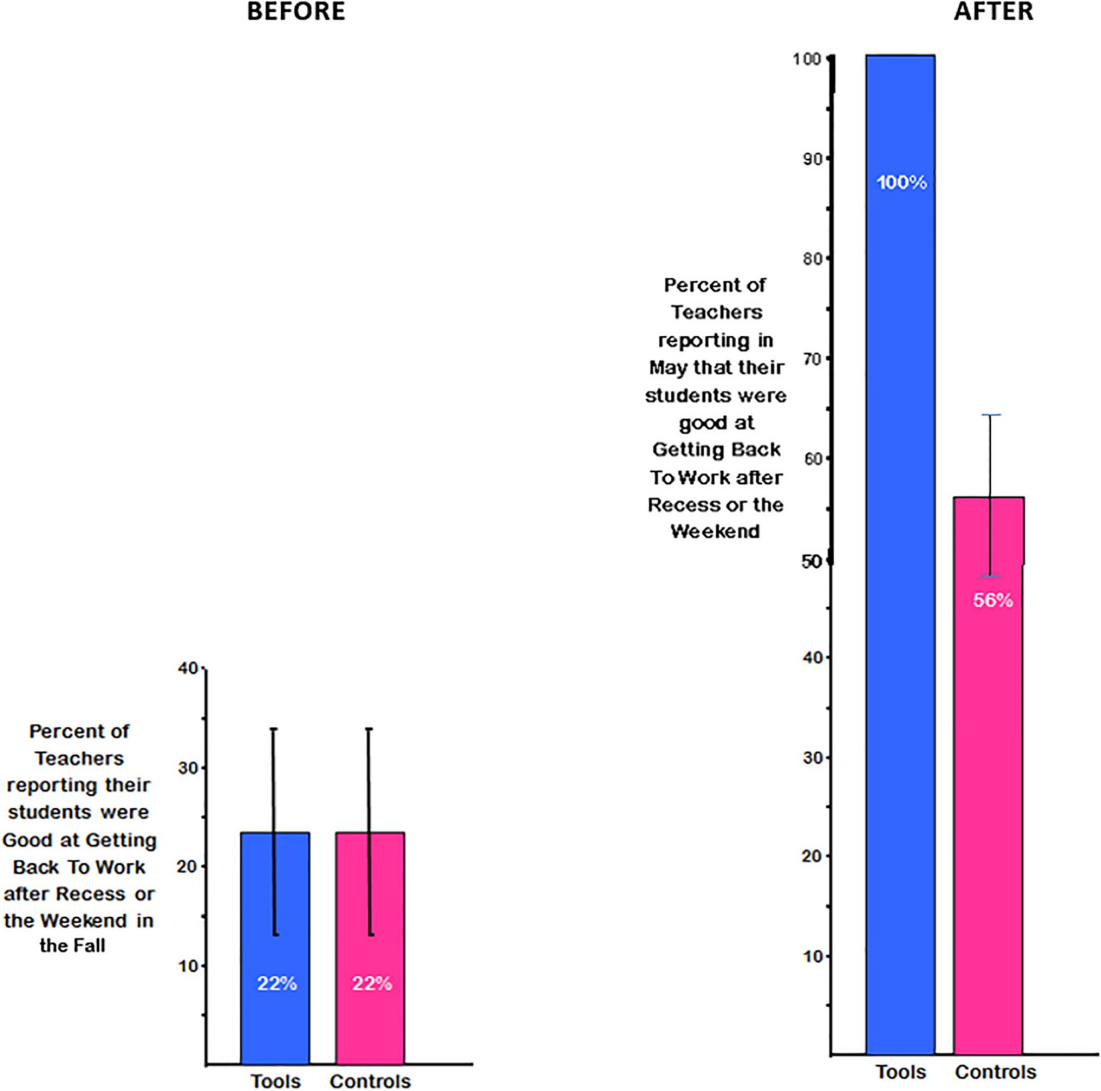
Teachers’ perceptions of students’ ability to get back to work after a break. In the Fall, few teachers thought their students were good at getting back to work after a break. By the Spring, Tools teachers were almost twice as likely as control teachers to think their children now were good at getting back to work after a break.

Back in the Fall, most teachers in both groups (89% in each) felt their students were *not* good at getting back to work after a break. Though comparable in the Fall, the groups differed by the Spring. ***All*** *Tools* teachers reported their students were good at getting back to work after recess and weekends; only 56% of control teachers reported that (χ^2^(1, N = 18) = 5.31, p < 0.02, odds ratio = 5.28; see Fig 5). Many *Tools* teachers mentioned how different this was from past years, e.g., “In 20 years I have never been able to come back from school holidays so seamlessly.” This difference already emerged by Spring break (χ2(1, N = 18) = 4.92, p = 0.02, odds ratio = 3.50). Eighty-nine percent of *Tools* teachers agreed strongly that their children had been good at getting back to work after Spring break; ***no*** control teacher strongly endorsed that. Indeed, 56% of control teachers disagreed, saying that their children had ***not*** been good at getting back to work after Spring break; only one *Tools* teacher disagreed.

#### Ability to work independently, without supervision

**Fig 6:**
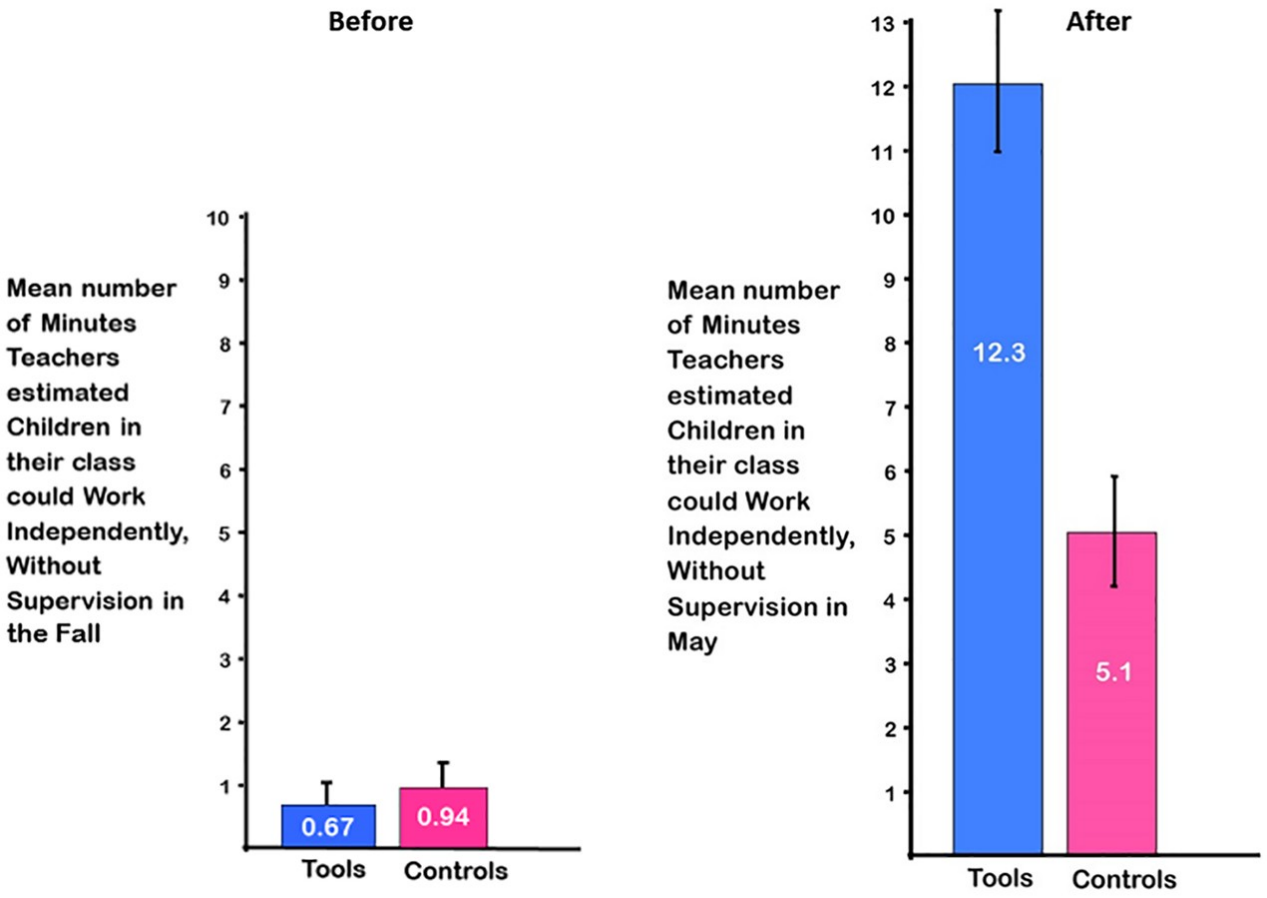
Ability of children to work on their own, unsupervised. By May, teachers in *Tools* felt their children could be left to work without supervision for far longer than did control teachers, although teachers’ estimates of this had been comparable in the Fall.

In the Fall, 55% of teachers in both groups said that children in their class were not capable of working on their own at all without supervision, even for a minute. The percentage of teachers endorsing that their children could work on their own for just 1–2 minutes without supervision was 44% for *Tools* and 33% for control teachers. Only one teacher said her students could work on their own for 3–5 minutes without supervision; that teacher was in the control group. By May, teachers in *Tools* said their children could be left to work without supervision for far longer than control teachers: F(1,15) = 11.43, p < 0.005, ηp^2^ = 0.43. See Table 2 and Fig 6.

**Table 2:** Teachers’ responses to the question, "If someone comes in your room now [in May], how long do you feel you could talk with that person and let the children in your class work on their own without supervision?".

Teachers’ estimates in May of how long children in their class could work on their own without supervision	Percent of *Tools* Teachers	Percent of Control Teachers
**> 15 min**	**22**	**0**
**11–15 min**	**33**	**0**
**9–10 min**	**33**	**11**
**6–8 min**	**11**	**33**
**3–5 min**	**0**	**33**
**≤** **1–2 min**	**0**	**22**

In their comments, teachers elaborated at length about this, and how different the experience this year in *Tools* classes was from previous years. See comments about this by *Tools* and control teachers in S5 File.

*Tools* teachers further commented on what the children’s better EF abilities to stay on task and control their attention and behavior has meant for what they can do in class: “The ability of my students to regulate their behaviour and to help those who still require some assistance has allowed me to be able to work with small groups as well as individually with specific students who require additional assistance. I have never been able to effectively do this ever with kindergarten students before.” “They are very self-regulated so I am able to work with a small group without being distracted. This is a wonderful gift.” “I have the freedom to work with small groups and help children learn at their own level; it helps provide students help where they need it and move them further faster. It is definitely more individualized…. Students easily work in small groups and can self-regulate while I work with students who need support.”

### Teachers’ feelings about teaching

**Fig 7:**
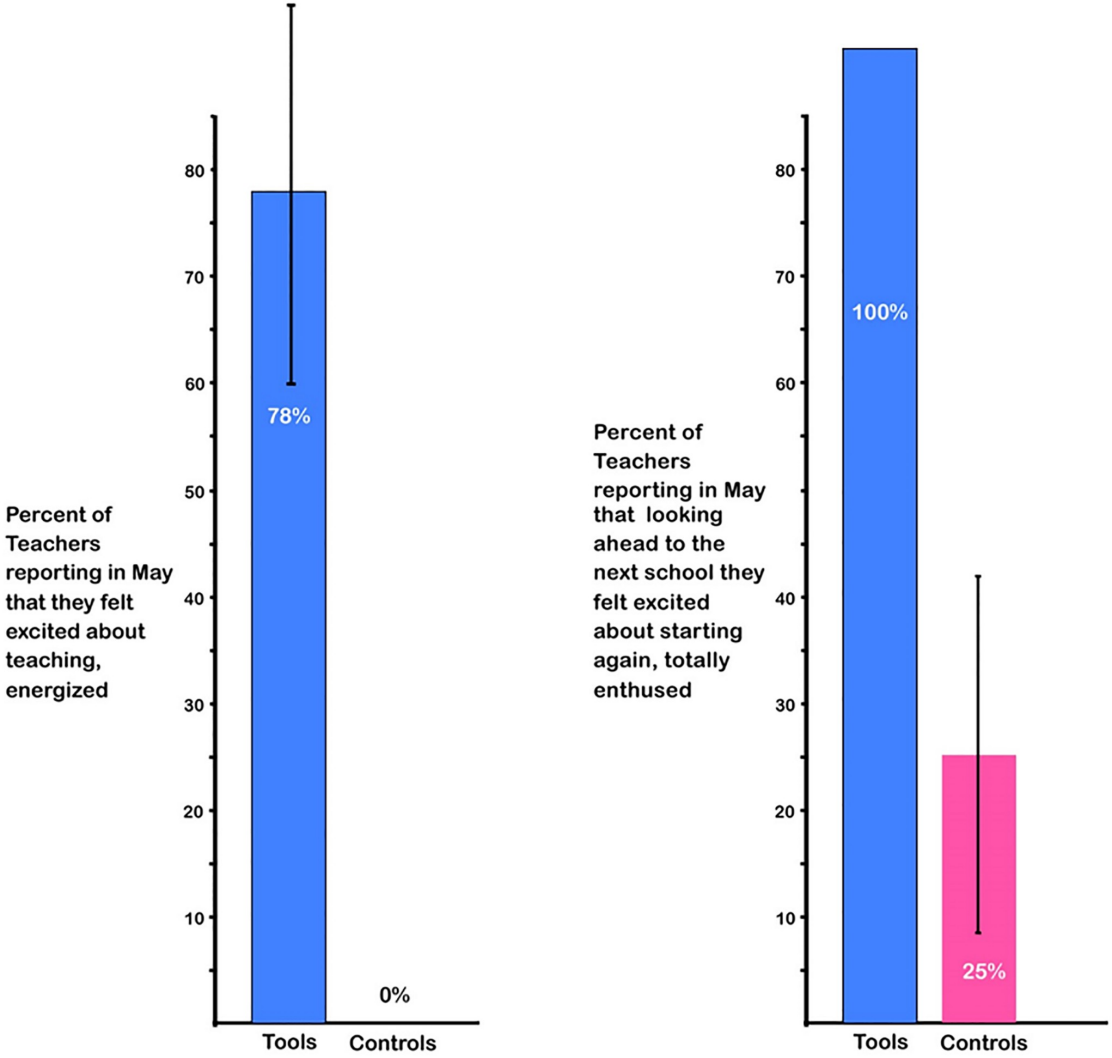
Teachers’ feelings about Teaching. Over three-quarters of *Tools* teachers, but none of the control teachers, responded in May that they were extremely excited about teaching (choices 1 or 2 on the 10-point scale): χ^2^(1, N = 18) = 4.99, p = 0.02, odds ratio = 3.58. Similarly, *all Tools* teachers responded that they were utterly enthused and looking forward to the next school year (choices 1 or 2 on the 10-point scale), while only 2 control teachers selected choice 2: χ^2^(1, N = 18) = 5.67, p < 0.02, odds ratio = 5.86.

We asked teachers to rate how they were feeling in May on a scale of 1 (excited about teaching, energized) to 10 (exhausted, burned out, weary) and to rate how they felt looking ahead to the next school year from 1 (excited about starting again, totally enthused) to 10 (not looking forward to it, looking forward to retirement). To both questions, over three-quarters of *Tools* teachers chose #1 or #2; ***no*** control teacher did. They were exhausted. See Fig 7.

Comments by *Tools* teachers indicated that students’ joy in coming to school, excitement about learning, and marked progress were the main contributors to their own excitement about teaching: “I have seen so much success in my students’ learning that I can’t wait to begin teaching again next year now that I have a better understanding of the program and all of its benefits!” “I have enjoyed seeing the students get so excited about coming to school and learning….[M]any students refused to miss school even if they were sick.” “What I have enjoyed most about my class this year is… The smiles and joy.” “What I liked most about teaching this year: Students’ enthusiasm towards learning and their pride in their development.” More comments by teachers are provided in S5 File.

### Change in teachers’ expectations of what kindergarten children are capable of

*Tools* teachers also expressed how their expectations for what the children could accomplish had changed, as had those of the parents: “Children in kindergarten are capable of so much more than I imagined.” “Parents are astonished with what their children can do.” “New kindergarten parents are pleased; parents who have had another child in kindergarten are amazed this year with what their children can do.”

## Discussion

This study found that *Tools* not only improves academic outcomes in reading and writing, but also shows for the first time that *Tools* also improves EFs in the classroom (being able to stay on task and quickly resume work after a break), markedly reduces teacher burnout and children being ostracized or excluded, and increases the joy students and teachers experience in school.

Limitations of the present study are: (1) Any new program may show benefits simply because it is new. *Tools* was new here and it was not compared to another new program but to a wait-list control group. (2) Teacher reports should be viewed cautiously because people can see what they hope and expect to see. (3) In the glow of the first year of a program, larger benefits are often seen than in subsequent years. (4) We might have had statistical power to detect group differences in math improvement or differential benefit from *Tools* in children from lower-income homes had we had more than nine classes per condition. (5) Though we had worked quite hard to match the *Tools* and non-*Tools* classes on teacher and student variables, hours of professional development, funds for materials, etc., one difference crept in unbeknownst to us: The *Tools* teachers arranged to meet together bi-monthly. It is possible that if the teachers in the control group had also met together, their results might have been better and the difference between their results and those from *Tools* less marked.

Even taking those considerations into account and therefore assuming that gains may appear larger here than they truly are, even if the true gains are half of what was observed, they are still quite impressive whether one looks at objective measures of academic performance, first-person reports of reduced teacher burnout, or teacher reports of student behavior. Results were better for *Tools* classes across most domains (reading, writing, peer inclusion, children’s ability to get along with, and be kind and helpful to, one another, children’s ability to work independently and stay on task without supervision, their ability to settle down quickly after a break and get back to work, teachers’ renewed joy in teaching, and students’ excitement about learning and joy in coming to school). The one exception was math, where results tended to be better for *Tools* classes, but not significantly so. The results were better than (a) the same teachers had in previous years and (b) control-group teachers had in the year of the study.

The percentage of children able to write one or more self-composed sentences was almost 300% greater in *Tools* classes (63% in *Tools* versus 22% in control classes), though writing skills had been comparable in *Tools* and control classes in the Fall. Conversely, the percentage of children whose writing skills were no better than the initial sounds of words was almost 300% greater in control classes (8% in *Tools* versus 23% in control classes). *Tools* emphasized writing and control classes did not. Thus, it is not surprising that children in *Tools* showed more advanced writing than control children. *The extent* to which children in *Tools* progressed is quite surprising, however—well beyond what anyone in BC public schools had seen previously in kindergarten children and well beyond the upper limits on the BC assessment tool for kindergarten. These results suggest that if writing is encouraged and supported in the way that *Tools* does, kindergarten children are capable of far more advanced writing than most educators and parents have assumed (and children start kindergarten 6 months younger in BC than in general in the US). (*Tools* teaches writing before reading, and being able to write is critical for recording what they plan to do in their play scenarios so children in *Tools* are highly motivated to master that. See S6 File for writing samples).

The developers of *Tools* had prioritized aligning *Tools* with Canadian learning standards and styles (this being the first implementation of *Tools* in any Canadian kindergartens) and had prioritized language skills over math skills for the first year of implementation. Thus, outcomes for language skills were markedly better for *Tools* versus control children and outcomes for math only marginally better. Importantly, math did not suffer in the *Tools* classes. There was no tradeoff with language skills being better in *Tools* classes and math skills worse. Math performance was at least as good, indeed marginally better, among children in *Tools* versus control classes.

By the end of kindergarten, *Tools* teachers estimated that their children could continue to work unsupervised for two and a half times longer than control teachers estimated for their students (12.3 versus 5.1 minutes). After breaks, 100% of children in *Tools*, but only about 50% of control children, could get back to work right away, according to teacher reports. This was echoed in marked differences between *Tools* and control classes in teachers’ comments about children’s self-regulation, ability to pay attention, and ability to work independently. This speaks to one of the greatest challenges voiced by Grade 1 teachers and one of their most common complaints—children’s poor self-regulation and ability to pay attention. Indeed, teachers report that the task of managing the classroom can lead to high levels of stress and burnout [85]. Children’s ability to pay attention when they enter Grade 1 predicts their later achievement in both math and reading [86, 87]. Children’s ability to work independently is critical to teachers’ ability to give individual attention to a student and for students to be able to work at different levels or follow their unique interests. Indeed, teachers mentioned that before *Tools* they had had difficulty supporting the more advanced students to move ahead of the rest.

In all control classes but one, teachers reported at least one child was likely to be left out by other children and in-group cliques had formed. In contrast, in all but two or three *Tools* classes, that was completely absent. Many control-group teachers mentioned in May that were still problems with some children hitting others or refusing to share, but that was no longer present in *Tools* classes. These findings have implications, we think, for reducing the incidence of bullying and of mental health issues in primary school.

Those large differences are particularly noteworthy because BC emphasizes educating children to be socially responsible citizens who are kind and compassionate. All teachers had this as a goal, but *Tools* enabled teachers to experience more success in realizing that goal, even though most teachers had received training in the *Second Step* social-emotional learning program and about half had received training in the *MindUp* mindfulness and social responsibility program. One would expect differences to be even greater between these *Tools* classes and classes where prosocial behavior was not a curricular priority.

Control group teachers were wait-list controls. They were looking forward to also being trained on Tools. In the meantime, during the study year, they were given the opportunity to get professional development workshops for free on whatever they wanted. They were thrilled with the three workshops they chose and much appreciated the funds we provided for new materials. There was no indication they were demoralized at not being chosen to be trained on Tools at the outset of this study.

By May, however, all teachers in the control group were indeed exhausted. *None* felt excited or energized about teaching or excited and enthused in looking ahead to teaching next year. In contrast, almost 80% of *Tools* teachers did (78% versus 0%). In part that was because the *Tools* teachers perceived their students as experiencing so much joy in school, progressing so far, and gaining so much confidence and sense of self-efficacy. Indeed, *Tools* teachers reported seeing improvements in all three core needs identified in self-determination theory [88] (increased feelings of social relatedness [community], autonomy, and perceived competence).

Economically-disadvantaged children benefited (as past studies had demonstrated, e.g. [68, 69]), but for the first time *Tools* was also tried in schools serving primarily middle and upper-middle income families. Children across the board benefitted from *Tools*–whether higher or lower socio-economic status (SES) and whether more advanced in academic skills or self-regulation at school entry or not. Outcomes did not differ significantly by teacher characteristics or children’s free-lunch or ESL status.

Children in *Tools* with weaker reading skills at school entry made greater progress in reading than other children in *Tools*. (Also, while the results for reading *had* differed across *Tools* classes with more versus fewer lower-income children in the Fall (F[1,6] = 5.74, p = 0.03, ηp^2^ = 0.24), those differences largely *disappeared* by the Spring (F[1,6] = 1.08, NS). *Tools*, thus, tended to reduce initial disparities separating children, schools, and teachers. Regardless of the SES levels of students in the class, the prevalence of English-language learners, or the experience level of the teacher, by May over half the children in *Tools* were able to read and write independently. Principals and resource teachers were surprised, when they walked into *Tools* classrooms in the Spring, to be unable to identify the special needs students.

Our assessment measures did not show greater writing gains for *Tools* children from lower-SES homes than from more economically-advantaged homes (χ^2^[1, N = 9] = 3.37, NS). Yet, when the 2 *Tools* expert trainers from Colorado came for their Spring workshop and were given de-identified samples of the children’s writing, to their surprise they were no better than chance at identifying those from low-income classes and those from middle-income ones, whereas in the Fall the differences had been stark. This suggests that while our measures did not indicate differential progress, the gap appears to have closed at least to some extent. Differences by SES were still present to be sure, but they were noticeably reduced (so much so that no significant differences remained).

Null results for *Tools* versus comparison conditions were reported at a Society for Research on Educational Effectiveness (SREE) conference [89, 90] although these have never been published in a peer-reviewed journal. At the SREE meeting, Lonigan [89] reported no differential benefit to EFs comparing preschool *Tools* to his program (*Literary Express*^™^). EFs were not assessed in that study, however, so the report of no difference in EFs outcomes is curious. Many of the schools in that study requested that their district adopt *Tools*; no school requested that for *Literacy Express*.

Wilson and Farran [90] reported null results from Year 1 of their study of pre-kindergarten *Tools* in Tennessee and North Carolina. Theirs was a textbook-perfect research design, but some of their outcome measures were prone to ceiling and floor effects (e.g., most 5-year-olds pass the Dimensional Change Card Sort test). One school district in the Wilson-Farran study was so impressed by the markedly better writing of *Tools* children that the district used its own funding to have all its teachers trained in *Tools*. (Assessment of writing had not been part of the research study). Other school districts that had been in the study did likewise because principals and kindergarten teachers felt they observed better social skills and readiness for learning in children who had attended *Tools* pre-kindergartens versus children from other pre-kindergartens. (The research study had not evaluated children in kindergarten, but only at the beginning and end of pre-kindergarten).

A limitation of the present study was the lack of funding to follow the children into Grade 1 and beyond, and also to include additional cohorts in teachers’ second and third years of implementing *Tools*, as had been our plan. We had hoped to investigate whether similar results would be replicated with other cohorts and to investigate how long gains would last and whether they might even increase. There is much evidence of academic gains increasing over time from *Tools* and from other beneficial programs (e.g. [68, 91–94]).

One reason particularly large effects may have been found in the present study is that all teachers in both the *Tools* and control group had indicated a willingness to learn and implement *Tools*. Other studies may have assigned teachers to *Tools* who might have been disinclined to implement it, weakening their effects. Usually teacher preferences are ignored in implementation studies. But teacher preferences can exert large effects! A teacher who is opposed to a program is less likely to do a good job implementing it [95–97]. After a study documents benefits from that program, that same teacher might then willingly implement it.

There are limitations on the possible applicability of the results found here to other contexts: (1) Important differences between early education in BC and the US led the developers of *Tools* to feel that the implementation of *Tools* in BC was more developmentally appropriate and a truer implementation of the *Tools* philosophy than *Tools* in the US. Without having to worry about high-stakes standardized tests at the end of year, stress levels were lower and *Tools* could be implemented the way it was intended–following each child’s lead. A particularly important difference to the developers of *Tools* was the stronger emphasis on play in BC *Tools* classrooms than in US *Tools* classrooms. In BC, children had an hour of play daily where they dramatized what they had been reading. They became deeply immersed in it, becoming the characters, and wrote about what they had learned about the lives of the people. Almost all children attained the level of intentional, mature make-believe play that Vygotskians associate with the development of self-regulation. In the US, because of the press for academics, children have only 20–30 minutes, and less as the year progresses, to dramatize stories and they do so only once a week instead of daily.

Clearly a full hour of dramatic make-believe play daily plus time each day for other types of play is not inconsistent with children doing extremely well in kindergarten, since the children in the present study did extremely well. Indeed, it is possible that copious playtime in kindergarten may be critical for laying the groundwork for academic success. This is especially noteworthy since there is enormous pressure on teachers to allow less and less time for play and devote more and more time to direct academic instruction, even in kindergarten [98, 99].

(2) Another limitation on possible generalizability is that most teachers in the present study were experienced. *Tools* is a demanding curriculum. Teachers in the present study bemoaned the amount of information to learn, e.g.: “The vast amount of materials that accompany the program is a challenge.” “The most challenging thing was implementing everything in the program. Adding a few new things was okay but having to learn everything and teach all new things at once was very challenging.” *Tools* may work best with teachers with at least a bachelor’s education, as most teachers here had.

This study does not enable one to determine “the active ingredient” of *Tools* nor which benefits of *Tools* contribute to making other benefits possible. Our hypothesis is that *Tools* works because of the gestalt that is *Tools* and that searching for the key element would be futile and fruitless. We hypothesize that *Tools* improves EFs because it directly trains, scaffolds, and challenges them, providing numerous opportunities to practice exercising them at progressively more advanced levels, *and* because it supports them by improving emotional and social well-being. We also hypothesize that *Tools* improves academic skills by directly targeting them in ingenious ways *and* because *Tools* improves EFs and emotional and social well-being.

Most elements of Tools probably affect more than one outcome. For example, scaffolding EF skills not only helps children practice those skills at a more advanced level than they would otherwise be able, thus aiding the development of those skills, but also reduces stress in the classroom. Teachers are less worried about the children not being able to exercise self-control or attention-regulation and children are less worried about being scolded for not exercising those EF skills [76, 100]. Conversely, stress impairs self-control [101, 102] and attention-regulation [50, 103], and reducing stress aids them. Also, by scaffolds increasing the likelihood of success and reducing the incidence of failure, they help build children’s self-confidence and belief in their ability to succeed [76].

Once children have a modicum of self-control and attention-regulation, that makes possible being able to work alone or with another child without the teacher needing to control the class from the front of the room. That makes possible a host of beneficial educational practices such as individualized pacing, instruction, and assessment because all the children doing the same activity together is no longer required [76, 100].

The paired play (pairing each child with every other at least once every week) not only helps each child get to know all the other children better and learn to get along with and work together with each (helping to build a sense of community), but also aids the development of language and EFs through the regulation of one another by verbal correction and feedback [80]. In Tools, each child in a pair gets to play the role of the “checker” and the one being checked, including younger children serving as the checker for older ones. The hands-on learning by working together with another child aids mastery of the academic material [76]. The improved sense of camaraderie in the classroom, which paired play facilitates, probably also aids EFs.

The clearest findings in the present study are: (a) *Tools* reinvigorated teachers’ enthusiasm for teaching. Those concerned with teacher burnout should take note. Burnout is leading many teachers to leave the profession [25, 104] and it causes many who stay to have less commitment to their job and less patience with the children [24, 104, 105]. Job burnout also contributes to poor health [106, 107]. Indeed, a one-unit increase in burnout score was found to be associated with greater risk for hospital admission for mental health problems and for cardiovascular problems [107]. (b) Teachers perceived *Tools* as making a big difference and perceived far better outcomes on an array of dimensions (academics; kindness, cooperation, and helping; joy in learning) than in the past. This seems to be a consistent theme across all studies of *Tools*, even where null findings have been reported. It is unlikely that that these findings are simply a halo effect for a new curriculum, because when compared head-to-head with another new curriculum, *Literacy Express*, in the study mentioned by Lonigan (89) many teachers requested that the district adopt *Tools* but none requested that for *Literacy Express*. It may be that teachers are seeing what they want or expect to see, but in the case of Diamond et al. [69] the teachers expected the district’s new program to yield better results than *Tools* and the district administration was very dismayed when it did not, since they had put so much effort into developing their new curriculum and were so proud of it. As researchers we need the humility to accept the possibility that teachers are picking up on things our assessment tools might be missing.

(c) *Tools* markedly improved reading and writing and these findings provide an existence proof that kindergarten children can write at more advanced levels than most had thought–composing sentences of their own creation with advanced vocabulary (e.g., stalagmites and stalactites). *Tools* teachers in the study said that their experience this year had changed their expectations of what kindergarten children could accomplish, e.g., “Children in kindergarten are capable of so much more than I imagined.” This occurred despite–or perhaps because of–carving out an hour a day for social dramatic play, encouraging other forms of play, and spending as much time on social-emotional growth as on academic growth. Clearly there is no indication whatsoever that play or social emotional learning interfered in any way with academic progress, and might well have aided it.

The findings of the study have relevance to several issues of keen scientific and societal interest: reducing the epidemics of bullying and teacher burnout, increasing student engagement in school, improving academic outcomes, and reducing socioeconomic inequalities in academic performance and EFs.

## Supporting information

S1 File *Tools* Brochure. (PDF)

S2 File BC’s kindergarten assessment tools. (PDF)

S3 File Survey monkey teacher questions. (PDF)

S4 File Table 3. All Dependent Measures analyzed, with Subsidized lunch, ESL, and Years Teaching as Covariates.
(DOCX)

S5 File Comments by teachers, parents, and principals. (PDF)

S6 File Two writing samples. (PDF)
