# Supplementary material for: Randomized control trial of Tools of the Mind: Marked benefits to kindergarten children and their teachers
Source: PLoS One. 2019 Sep 17;14(9):e0222447. doi: 10.1371/journal.pone.0222447 (PMC6748407; doi:10.1371/journal.pone.0222447)
Supplement: S1 File — (PDF) [file pone.0222447.s001.pdf]

TOOLS OF THE MIND

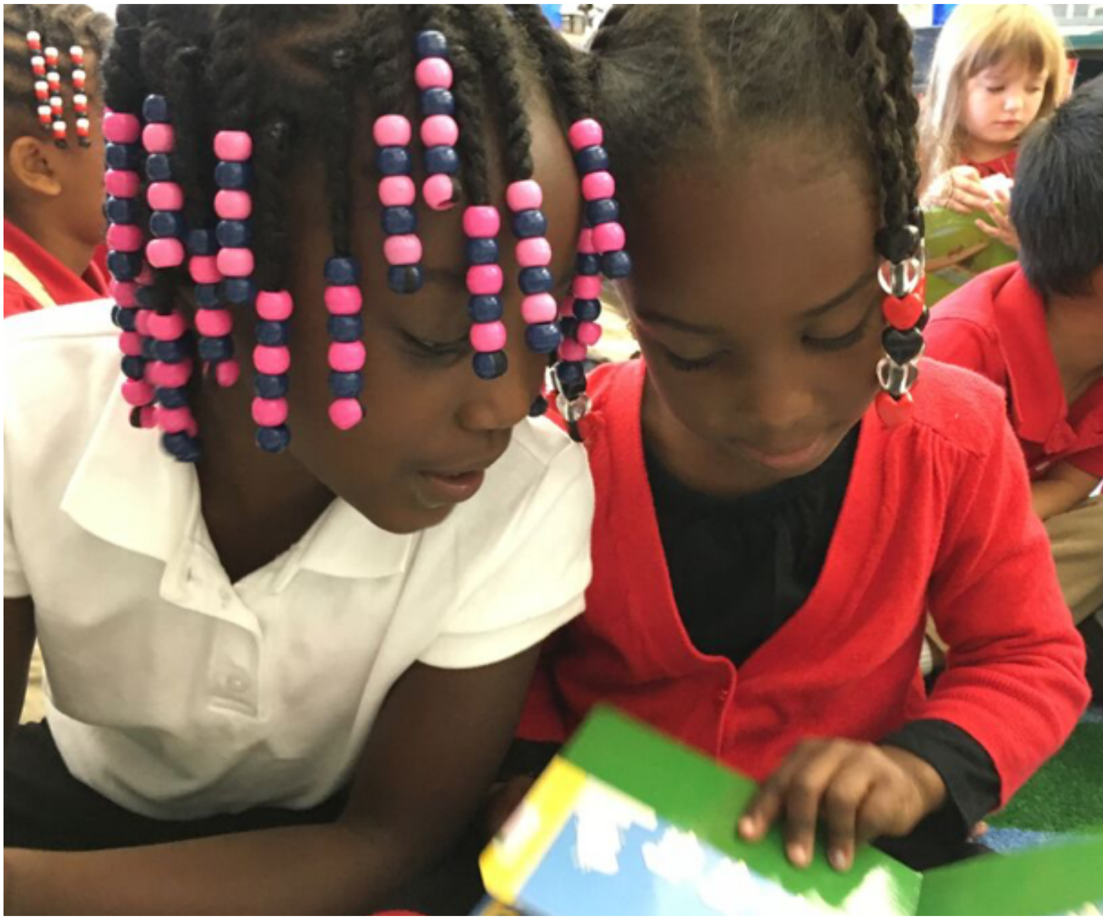

K

What makes Tools unique?

In order to learn in elementary school and be successful students, children must develop the ability to self-regulate. Self-regulation encompasses not only emotions, physical behavior and social interactions, but includes the ability to monitor and control cognitive processes such as attention.

It includes:

- ✦ *The ability to stay on task*
- ✦ *Ignore distractions*
- ✦ *Remember on purpose*
- ✦ *Hold two strategies in mind at the same time*
- ✦ *The development of self-discipline*
- ✦ *The motivation to succeed*

Even though children may know many facts, without self-regulation they will not learn new things efficiently and will have trouble with more-advanced content.

In school, children need to conform to routines, switching from doing something they enjoy to something of lesser interest. They are expected to be able to follow directions with multiple interrelated steps and are required to control their attention by blocking out distractions. They have to be able to work with other children and focus on the learning task.

> Our philosophy is that teaching should be more than transmitting facts and skills; it should teach children about learning itself, giving them the mental tools that will enable them to learn on their own.

Self-regulation is what allows children to be successful in meeting these demands.

When children are self-regulated, every activity they engage in is a learning activity and they can follow rules without the teacher's support. Children who are not self-regulated can behave *if* the teacher is watching them and learn *if* the teacher is helping them pay attention, but without the teacher's direction, the child will not (cannot) learn. Moreover, when teachers have to spend significant time regulating children in their classrooms, they have less time and attention to invest in supporting children's learning of academic skills, and challenging and individualizing instruction for all students.

In a survey of kindergarten teachers, 46 percent reported that more than half of their children do not have sufficient self-regulation. (Rimm-Kaufman, 2000.) Helping young children improve their self-regulation is critical to closing the achievement gap for many at-risk children, as well as helping all children reach their highest potential.

The good news: Early childhood is a key period for acquiring self-regulation in all its forms. This is why in a Tools of the Mind Kindergarten, teachers work deliberately to help children develop self-regulation.

> “All children have to learn to be self-regulated. Teacher-regulation is not sufficient for the development of self-regulation.”

How does a focus on self-regulation translate to the kindergarten classroom?

The central focus of Tools of the Mind (Tools) is the development of both cognitive and social-emotional self-regulation *at the same time* that academic skills are taught. In Tools, the focus on self-regulation is not limited to a "stand alone" activity, but is embedded into the Tools curriculum. In a Tools Kindergarten:

- ✦ Practice in self-regulated learning is embedded into all activities.
- ✦ Teachers use mature intentional dramatic play as an activity to help children develop important underlying cognitive skills.
- ✦ Teachers emphasize the application of self-regulation to learning itself, facilitating self-regulation development in specially designed learning tasks.
- ✦ Research-based literacy and math activities are modified to include self-regulatory components.
- ✦ Specific instructional activities are designed to teach self-regulation and reflective thinking.
- ✦ Classroom management techniques maximize productive interactions and task involvement.

What is it like to be a Tools kindergarten teacher?

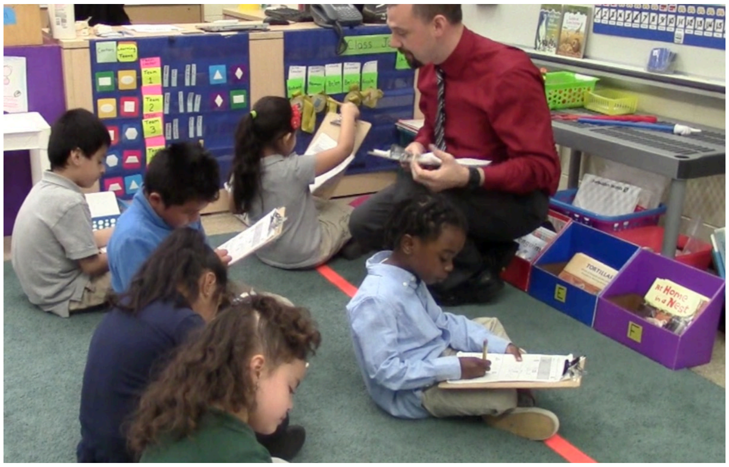

In Tools, the process of learning is as important as the content that is to be learned. Children in Tools Kindergartens use learning plans, work in "Study Buddy" pairs, engage in learning games and conference weekly with teachers to discuss their learning. The fall Kindergarten classroom activities are different from the spring activities to match both the design and content of activities to children's developmental needs and goals. Tools instructional interactions are planned to scaffold each child and to help teachers be more effective in identifying specific teachable moments. Tools teachers focus on helping children become intentional and reflective learners, creating a classroom in which instruction in literacy, mathematics and science reflects children's learning capacity, rather than age-level expectations.

Tools kindergarten teachers:

- ✦ **Develop** an understanding of scaffolding and individualization that reflect Vygotskian principles of teaching and learning.
- ✦ **Target** their interactions to children's individual zones of proximal development (ZPD).
- ✦ **Assess** children's learning and development on a daily basis to inform instructional decisions.
- ✦ **Know** the developmental progression of learning tasks and use this knowledge in their decision-making.
- ✦ **Recognize** that the kind of assistance they provide is critical because it affects when and how the child will eventually be able to perform a task independently.
- ✦ **Possess** the ability to provide the right level of scaffolding and maintain it at a level that fits the child's emergent competencies.
- ✦ **Know** how to use tactics like mediators, private speech, written language and shared activity to support learning.
- ✦ **Facilitate** children's development of a set of "Mental Tools," helping children become masters of their own learning.
- ✦ **Create** a classroom where children who are working independently are practicing skills correctly and in appropriate ways.

Examples of Tools K Activities

Graphics Practice

In Graphics Practice, children develop fine motor skills, practice letter formation and develop the penmanship and self-regulation skills needed for writing. They draw on white boards with markers, stopping and starting in response to musical cues. Children use private speech to help them remember how and what to write, learning to inhibit while also remembering the shape they're representing in writing.

Elkonin Boxes I and II

In Elkonin Boxes I and II, small groups of children jump on carpet squares, use a specific gesture or move symbolic tokens as they separate out the sounds in words looking at specially designed Elkonin Box cards that visually represent phonemes in the names of pictures like "cat." Children focus attention on specific parts of a word, use mediators and private speech while developing phonemic awareness and practicing the alphabetic principle.

Venger Drawing

In small groups, teachers help children plan and discuss various ways to incorporate a geometric shape into a drawing. Children use geometric terms and positional vocabulary, brain-storming possibilities from multiple perspectives. Children each verbalize a plan for their drawing and create a unique representation incorporating the geometric shape.

Learning Conferences

Children meet 1:1 with their teacher to set new learning goals and discuss their work habits, how they learn and any difficulties with concepts and skills. Teachers emphasize learning how to help yourself remember, practice effectively and stay motivated even when things are frustrating.

Venger Word Problems

In a collaborative partnered mathematics activity, children solve word patterns that require logical thinking with the aide of a number line and other mediators.

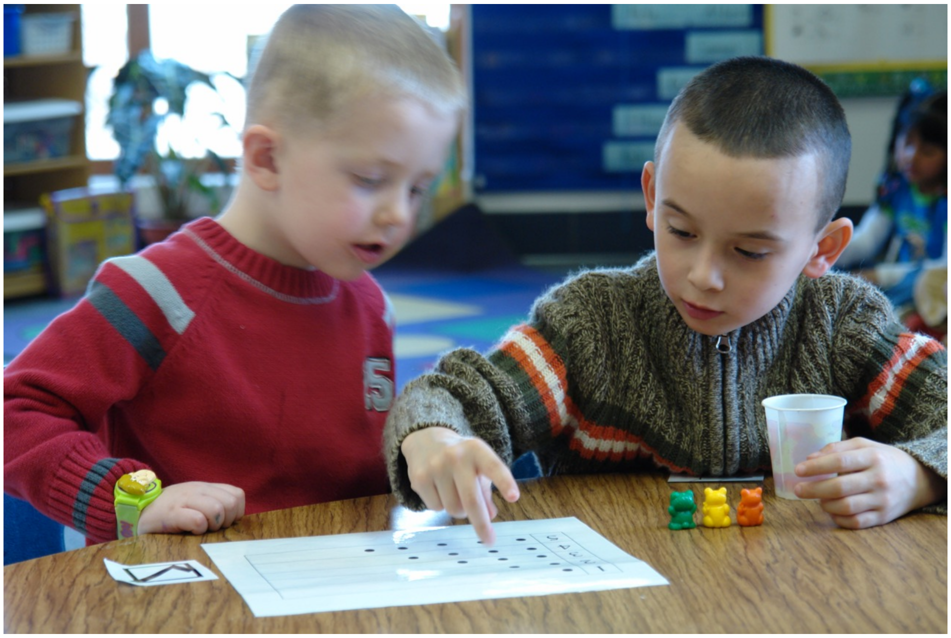

Characteristics of Tools of the Mind

- Tools is a comprehensive curriculum including content that meets Common Core State Standards. Content is presented in an integrated, developmental way so that instruction forms a coherent whole.
- The instructional formats and activities in Tools are research based.
- Instructional strategies used in Tools include child-directed activities, teacher-directed activities and collaborative partner activities.
- The thoughtful combination of instructional strategies and the matching of instructional strategy to activity is specifically designed to support self-regulation development and allow individualized instruction in academic skills.
- Individualization through multiple levels of scaffolding and on-going use of assessment data to tailor interactions to meet individual needs is central. Progress is monitored daily, weekly and monthly.

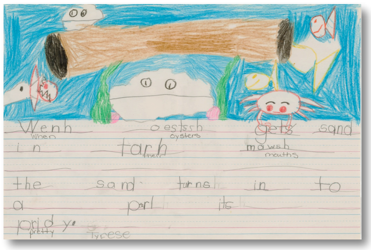

How Tools is implemented

- Tools is designed as a core curriculum that works for all children including those with identified special needs.
- Tools activities are multi-level so instruction is individualized within the design of each activity. Individualizing instruction happens within all activities.
- Teachers learn specific scaffolds to support children's development in all activities.
- Tools provides literacy benchmarks, benchmark assessments and tracking support and guidance about how to meet the needs of children below, at and above benchmarks.
- Pacing Guides provide guidance to teachers about how to increase challenge across the year, ensuring that instruction matches end-of-year district benchmarks and goals.
- A pair of Materials Kits with high usage materials are available for purchase through Lakeshore. Tools provides book lists for coordinated core readers. Consumable and activities templates are available to contracted districts for free download from password protected eTools webpage.

www.toolsofthemind.org

Evidence of Effectiveness

A randomized control design study (2014) found that Tools closed the achievement gap for at risk children, with positive effects on executive functions, reasoning, control of attention, reading, vocabulary and math. These effects extended into First Grade, with children from Tools classrooms learning at a faster rate than children from control classrooms. As well, significant effects on children's stress response physiology was seen in Tools of the Mind classrooms with a high percentage of at risk children.Tools classrooms were found to have a high level of emotional and instructional support regardless of children's social-economic level, eliminating the effects of poverty on classroom climate.

In another study, children in Tools classrooms were found to have higher rates of self-regulation in a National Institute for Early Education Research (NIEER) double-randomized study. This study compared children in Pre-K Tools classrooms with a control group using a high-quality ECE program with no emphasis on self-regulation. In addition to student gains, teachers trained in Tools scored higher in classroom management measures, used classroom time more productively and had a higher rate of appropriate and cognitively challenging interactions, as measured by the Early Childhood Environmental Rating Scale and the CLASS.

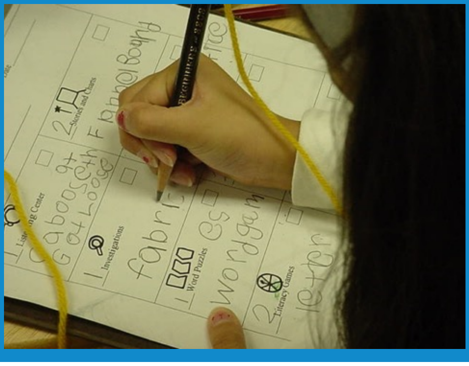

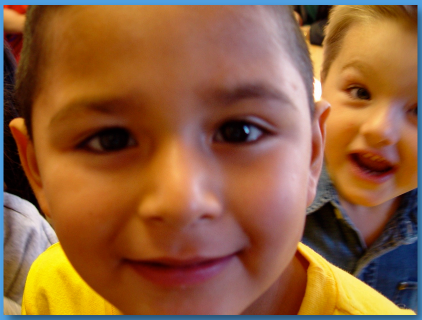

Professional Development

District capacity building is an important aspect of Tools training. Professional development is a two-year process. At the end of the two years, districts have a strong core of teachers who understand and can apply the theories that shape Tools of the Mind, with a new set of teaching skills and instructional strategies. The Tools professional development process is designed to have flexibility and responsiveness built in. Tools staff collaborate with districts to design the details of each district's professional development plan. Our professional development resources offer a range of ways to support adult learners––from in-person or virtual workshops to teacher materials on our password protected eTools section on our website, to the iScaffold app with video and Quick Start Guides for activities available on iPad or via a web browser. Teachers receive responsive, flexible and ongoing support through virtual and in-person site visits and iScaffold.

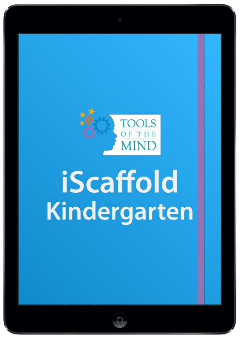

Year 1

In the first year, professional development rolls out in four phases, matching the developmental trajectory of child skills across the year. Teachers receive responsive, flexible and ongoing support through site visits and iScaffold.

Year 1 professional development workshops emphasize classroom management, self-regulation development, and literacy, math and science content areas.

Year 2

Year 2 professional development is designed to help teachers become more intentional in applying the underlying theory behind the program to their unique students and context. Core classroom practices, key steps in activities to support self-regulation development, and dynamic assessment and individualization of instruction are central focuses.

> “The strength of the Tools approach to professional development is the consistent focus on improving teacher performance through embedded technical assistance visits. The visits are more than one-stop meetings. Rather, they are visitations where consultants are available and willing to answer all questions from any member of the staff.”

Continuing Education

After the two years of Core Training, Tools of the Mind partners with programs to build Communities of Practice providing opportunities for teachers to collaborate with colleagues in the region and leave with action plans to target continued development. Single-day workshops focus on new topics, from social skills & social-emotional regulation development, to technology & interactive media in the Tools classroom, offering teachers new materials and ideas, and the chance to dig into discussions about how to further improve child outcomes in their classrooms and communities.

For more information

Phone: 720-541-9597
/ToolsoftheMind
@Tools_Mind
Web: www.toolsofthemind.org

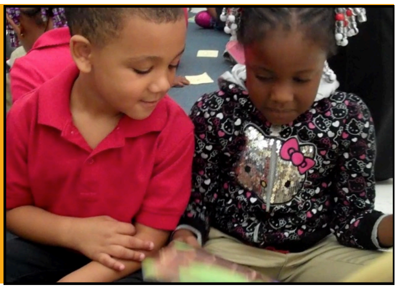

National and International Recognition

UNESCO

In 2001, the International Bureau of Education, an arm of the United Nations Educational, Scientific and Cultural organization (UNESCO), named Tools an exemplary innovative educational program.

Others

Footage showing Tools classroom activities can be seen in the "Heads-up Reading" television series and the "Growing and Learning in Preschool" video produced by the National Institute for Early Education Research (NIEER). Scaffolded Writing, a technique invented by Tools to teach writing, has been named as a model literacy technique by the International Reading Association.

Further Reading

Blair C, Raver CC (2014) Closing the Achievement Gap through Modification of Neurocognitive and Neuroendocrine Function: Results from a Cluster Randomized Controlled Trial of an Innovative Approach to the Education of Children in Kindergarten. PLoS ONE 9(11): e112393. doi:10.1371/journal.pone.0112393 [Link]

S. Barnett, K. Jung, D. Yaroz, J. Thomas, A. Hornbeck. "Educational effects of the Tools of the Mind Curriculum: A randomized trial." *Early Childhood Research Quarterly,* March, 2008. [Link]

Diamond, S. Barnett, J. Thomas, and S. Munroe. "Preschool Program Improves Cognitive Control." *Science*, November 2007. [Link]

E. Bodrova and D. J. Leong. *Tools of the Mind* (2nd edition). Merrill/Prentice Hall, 2007.

E. Bodrova and D. J. Leong. "The Development of Self-Regulation in Young Children: Implications for Teacher Training." In M. Zaslow & I. Martinez-Beck (Eds.), *Future Directions in Teacher Training.* Brooks-Cole (p. 203-224), 2006.

E. Bodrova and D. J. Leong. "Vygotskian Perspectives on Teaching and Learning Early Literacy." In D. Dickenson & S. Neuman (Eds.), *Handbook of Research in Early Literacy Development* Volume 2, Guilford Press (p. 243-256), 2006.
