# Supplementary material for: Randomized control trial of Tools of the Mind: Marked benefits to kindergarten children and their teachers
Source: PLoS One. 2019 Sep 17;14(9):e0222447. doi: 10.1371/journal.pone.0222447 (PMC6748407; doi:10.1371/journal.pone.0222447)
Supplement: S2 File — (PDF) [file pone.0222447.s002.pdf]

Early Learning Framework

Early Learning Projects

Primary Years

Play Today

StrongStart BC

Teaching and Assessment Tools

Reading for Families

Ready, Set, Learn

Training and Professional Development

Teaching and Assessment Tools

Kindergarten Learning Project

Use these field-developed and tested teaching and assessment materials to measure children's learning and development in the Kindergarten program.

Oral Language

Oral language provides the foundation for all literacy development. Talking about experiences and ideas builds the concepts used later in reading, writing, and numeracy.

- Oral Language Assessment Continuum (PDF)

Sample Assessment Profiles

- Oral language individual assessment 1 (PDF)
- Oral language individual assessment 2 (PDF)
- Oral language individual assessment 3 (PDF)
- Oral language class tally (PDF)

Social Responsibility

Learning is an interactive process for young children, and the development of social responsibility goes hand-in-hand with oral language development.

- Social Responsibility Assessment Continuum (PDF)

Sample Assessment Profiles

- Social Responsibility individual assessment 1 (PDF)
- Social Responsibility individual assessment 2 (PDF)
- Social Responsibility individual assessment 3 (PDF)
- Social Responsibility class tally (PDF)

Reading and Viewing

Kindergarten children develop as early readers through many experiences with

different forms of text in a print-rich environment.

- Reading and Viewing Assessment Continuum (PDF)

Sample Assessment Profiles

- Reading and Viewing individual assessment 1 (PDF)
- Reading and Viewing individual assessment 2 (PDF)
- Reading and Viewing individual assessment 3 (PDF)
- Reading and Viewing class tally (PDF)

Writing and Representing

As young learners begin to comprehend printed material, they express their ideas in a variety of forms that often combine drawing, abstract symbols and oral explanation.

- Writing and Representing Assessment Continuum (PDF)

Sample Assessment Profiles

- Writing and Representing individual assessment 1 (PDF)
- Writing and Representing individual assessment 2 (PDF)
- Writing and Representing individual assessment 3 (PDF)
- Writing and Representing class tally (PDF)

Numeracy

Early numeracy grows as children explore the everyday world of shape and space, patterns, and numbers through hands-on materials, games and number activities.

- Numeracy Assessment Continuum (PDF)

Sample Assessment Profiles

- Numeracy individual assessment 1 (PDF)
- Numeracy individual assessment 2 (PDF)
- Numeracy individual assessment 3 (PDF)
- Numeracy class tally (PDF)

Student Portfolio

Student profiles and portfolios are useful in discussions with families, other educators, or resource persons involved in supporting student growth. Teachers create profiles and portfolios by recording observations on the assessment continua

at several different points in the year. They also gather work samples and photographs that illustrate and supplement this information. A simple one-page summary sheet for organizing a student portfolio is provided in this section, along with examples of completed student profiles and portfolios.

- Learning Profile and Portfolio Template (PDF)

Sample Portfolio Documents

- Oral Language Portfolio 1 (PDF)
- Oral Language Portfolio 2 (PDF)
- Oral Language Portfolio 3 (PDF)

More Assessment Info

Explore how a child's progress is assessed once they are in the Kindergarten to Grade 12 school system.

- Assessment profiles for educators

Contact Information

For more information, please contact the Early Learning Team:

Email:
EDUC.EarlyLearning@gov.bc.ca

Assessment Instrument Table: DRA2

Element	Description	
Instrument Name	Name of specific instrument (more than vendor name).	Developmental Reading Assessment 2nd edition (DRA2)
Vendor	Name of the company or organization that produces the instrument.	Pearson
Purpose (Intended Use)	The described purpose and appropriate uses of the instrument.	**DRA2** enables primary teachers to systematically observe, record, and evaluate changes in student reading performance. DRA2 provides teachers with information that helps teachers determine each student's independent reading level and identify what the student needs to learn next.<br><br>The **DRA Word Analysis** is a diagnostic assessment that provides classroom and reading teachers with a systematic means to observe how struggling and emerging readers attend to and work with the various components of spoken and written words. It is intended to support teachers to:<br>1. Determine students' level of control of various word analysis tasks.<br>2. Document students' progress over time.<br>3. Group students according to their instructional needs.<br>4. Plan more effectively for instruction.
Population	Who (which students) could be assessed using the instrument.	DRA2 can be used with students from kindergarten through eighth grade. It includes a K-3 kit and a 4-8 kit.<br><br>The DRA Word Analysis is intended for:<br>• Emerging readers in kindergarten and beginning first grade to identify their level of phonological awareness and basic knowledge of phoneme/grapheme relationships.<br>• Struggling readers in the latter part of first grade through third grade who are reading below grade level or designated levels of proficiency due to ineffective word-solving skills and strategies.<br>• Fourth- and fifth-grade students whose independent DRA reading level is 38 or below.
When? How frequently?	How frequently the instrument can be administered in a school year, and	The DRA2 assessment can be used on a semi-annual or annual basis to monitor and document change over time in each student's reading. It may be used more frequently with

	recommended or required administration windows.	struggling readers to ensure continued progress. Testing windows are set at the local level. CDE encourages districts to administer DRA2 at least three times during the school year.<br><br>**DRA Word Analysis** should be administered during the first part of the school year after the DRA2 has been administered to students in first through fifth grades. Teachers will use the information gained from the DRA2 to determine which emerging and/or struggling readers should be given this assessment. It is best to wait until midyear to give this assessment to emerging readers in kindergarten. It is also recommended that teachers re-administer the DRA Word Analysis midyear and at the end of the school year to:<br>• Determine if students have gained control of those tasks that they initially demonstrated no, little, and/or some control.<br>• Identify a new focus of instruction for students who are still reading below a designated level of proficiency on the DRA2 due to ineffective word-solving skills and strategies.
Content Area (s)	Content area or areas being assessed.	Reading
Learning Objectives	Specific learning objectives being assessed, at as detailed a level as is provided. This may be "topics" or categories or may be actual learning objective statements.	**DRA 2**<br>Reading engagement (student survey) -- describes the student's level of engagement with reading. Engaged readers read often, know books and authors, and have goals for themselves as readers.<br><br>Oral reading fluency (student oral reading of text at an appropriate level)<br><br>Comprehension (retell, responding to comprehension questions, write summaries)<br><br>**DRA Word Analysis**<br>1) Phonological awareness: rhyming, alliteration, phonemic awareness, and segmentation<br>2) Phonics: encoding, decoding, substitutions/analogies<br>3) Meta-language (language used to talk about printed language concepts)<br>4) Letter/Word Recognition<br>5) Structural Analysis and Syllabication
Individual Metrics	The scores provided at the individual (student) level.	**DRA2 Scores:**<br>• Students receive individual scores for reading engagement, oral reading fluency and comprehension/printed language concepts. Depending on the student's independent

reading level, scores are translated into a performance level of: intervention, instructional, independent, or advanced.

- Students oral reading fluency and comprehension/printed language concepts scores are combined to determine an *overall performance level*, which also depends on the students independent reading level. Overall performance level ratings include: Emerging, Developing, or Independent for Levels A–12; and Intervention, Instructional, Independent, or Advanced for Levels 14–40.
- Students also receive an independent reading level rating from level A to level 40 (A, 1, 2, 3, 4, 6, 8, 10, 12, 14, 16, 18, 20, 24, 28, 30, 34, 38, and 40). Independent Reading Level is the reading level at which the student can engage with the text independently (e.g., the teacher does not provide any scaffolding). The student's total score in Oral Reading/Oral Reading Fluency and the student's total score in Comprehension/ Printed Language Concepts determines whether a text was read at an independent, instructional, or advanced level.

The relationship between student performance ratings and independent reading levels are described in greater detail below:

Reading Engagement: Teachers rate students' responses in the Student Reading Survey. Scores range from 2 to 8, where scores of 2 to 3 indicate an Intervention level of performance, scores of 4 to 5 indicate an Instructional level of performance, scores of 6 to 7 indicate an Independent level of performance, and a score of 8 indicates an Advanced level of performance.

Oral Reading Fluency: At levels 14-80, Oral Reading Fluency describes the student's oral reading behaviors in terms of expression, phrasing, rate, and accuracy. At levels 4-12, Oral Reading Fluency is comprised of phrasing, monitoring/self-corrections, problem-solving unknown words, and accuracy. The Oral Reading Fluency score is the sum of the four indicators (e.g., for levels 14-80, Expression, Phrasing, Rate, and Accuracy). Scores range from 4 to 16, where scores of 4 to 6 indicate an Intervention level of performance, scores of 7 to 10 indicate an Instructional level of performance, scores of 11 to 14 indicate an Independent level of performance, and scores of 15 to 16 indicate an Advanced level of performance.

Comprehension/ Printed Language Concepts:
Comprehension describes the student's ability to retell and understand the text including the main ideas, key facts, and characters, events, or topics. At lower levels (A- 3), printed language concepts are evaluated. At Levels A–1, the student's use of printed language concepts is evaluated, specifically directionality and one-to-one correspondence. At Levels 2–3, evaluation of students' use of words/letters is added. At Levels 4–24, in addition to evaluating the student's retelling of the story (including the sequence of events, characters and details, and key vocabulary), the teacher evaluates the student's preview or predictions about the story, the level of interpretation of the story, the level of reflection on the story, and how much teacher support the student required to retell the story. At Levels 4–16 only, a student's performance is evaluated for making connections with the text. At Levels 28–80, teachers rate the student's responses to the questions and prompts in the Student Booklet. At Levels 28–38, teachers also evaluate the use of key vocabulary in the summary. At Levels 40–80, teachers additionally evaluate the skill of Metacognitive Awareness. Each task is rated on a four-point scale. Different descriptions are used for fiction and nonfiction texts for Summary and Reflection and also for texts at Levels 28– 38 versus Levels 40–80. The teacher selects the best description of the student's performance on each indicator and sums the score to obtain the Comprehension score. Comprehension scores range in DRA2 K–3 from 7 to 28 (except Level 40, which ranges from 6–24); and in DRA2 4–8, scores range from 6 to 24.

With DRA2 K–3 (except Level 40), scores of 7 to 13 reflect an Intervention level of performance; scores of 14 to 18 reflect an Instructional level of performance; scores of 19 to 25 reflect an Independent level of performance; and scores of 26 to 28 reflect an Advanced level of performance. For independent reading level 40, scores of 6 to 11 indicate an Intervention level of performance, scores of 12 to 16 indicate an Instructional level of performance, scores of 17 to 22 indicate an Independent Level of performance, and scores of 23 to 24 indicate an Advanced Level of performance.

DRA Word Analysis
A total of 40 tasks are available. Each task produces a raw score (range of 7-50), which can be categorized into four levels of control:

- No/Little Control (0-39% correct)
- Some Control (40-79% correct)

		• Gaining Control (80-99% correct)<br>• Control (100% correct)<br>Testing stops when the student is no longer able to perform well on any three tasks (i.e., does not demonstrate control).
Individual Comparison Points (cut scores)	Information provided regarding how good is good enough performance on the instrument. Comparison information should be available for every individual metric. This may be performance level ratings with specific cut scores.	Students **reading engagement**, **oral fluency**, **comprehension/printed language concepts** are rated at four levels: Intervention, Instructional, Independent and Advanced. Students rated at an "independent" or "advanced" level are considered proficient.<br><br>**Independent Reading Level Expectations:**<br>Note: the spring reading level is the end of year expectation for each grade level.

Grade	Time	Proficient/Independent	Instructional
Kindergarten	Fall	Pre A	
	Mid-Year	1	A
	Spring	3	2
1st Grade	Fall	3	2
	Mid-Year	8	6
	Spring	16	14
2nd Grade	Fall	16	14
	Mid-Year	20	18
	Spring	28	24
3rd Grade	Fall	28	24
	Mid-Year	34	28
	Spring	38	30-34
4th Grade	Fall	38	30-34
	Mid-Year	38 (34-39)	34
	Spring	40 (28)	38
5th Grade	Fall	40 (28)	38
	Mid-Year	40 (34-36)	38
	Spring	50 (28)	10
6th Grade	Fall	50 (28)	10
	Mid-Year	50 (33)	10
	Spring	60 (28-30)	50
7th Grade	Fall	60 (28-30)	50
	Mid-Year	60 (32-34)	50

	Spring	70 (31-33)	60
8th Grade	Fall	70 (31-33)	60
	Mid-Year	70 (35-36)	60
	Spring	80 (31-32)	70

**Individual Comparison Points (CDE)**	Cut Scores for Significant Reading Deficiency	CDE has identified the following cut scores for students *independent reading level* as scored by DRA2. Students scoring at the identified independent reading level or lower would be identified has having a significant reading deficiency.

	**Fall**	**Winter**	**Spring**
Kindergarten	**NA***	**NA****	**A**
1st Grade	**A**	**6**	**10**
2nd Grade	**10**	**14**	**18**
3rd Grade	**18**	**20**	**28**

*Kindergarten: For the beginning and middle of the year, teachers should use the Word Analysis assessments from DRA2 for Kindergarten students to determine a Significant Reading Deficiency. For the beginning of the year, teachers should use Tasks 3, 5, and 6 (Isolating Initial Sound, Recognizing Lowercase Letters, and Recognizing Capital Letters). Students should score higher than 3 on Task 3 (Isolating Initial Sound) and higher than 9 on Tasks 5 and 6 (Recognizing Lowercase and Capital Letters). Students must score above the cut-off score on at least one of the three tests to not be identified as having a Significant Reading Deficiency.
**Kindergarten: For the middle of the year, in addition to Tasks 3, 5, and 6, teachers should use Task 21 (Segmenting Words into Phonemes). Students should score higher than 7 on Task 3 (Isolating Initial Sound), higher than 20 on Tasks 5 and 6 (Recognizing Lowercase and Capital Letters), and higher than 5 on Task 21 (Segmenting Words into Phonemes). Students must score above the cut-off score on at least one of the four tests to not be identified as having a Significant Reading Deficiency.

Aggregate Metrics	Scores provided at the group level. The group could be a grade level, school, district, or disaggregated groups (e.g. race/ethnicity, gender, IEP status, FRL status) Specify the group(s) and the score(s) provided.	• The number and/or percent of students reading at the independent or advanced level for the expected independent reading level (by grade level)<br>• The number and percent of students identified as having a significant reading deficiency (by grade level)

Aggregate Comparison Points (cut scores)	Information provided regarding how good is good enough performance at the group level.	None provided by vendor.
**Aggregate Comparison Point (CDE)**	CDE has established comparison points for requests to reconsider	50% of students receive an independent or advanced over-all performance level rating for the target independent reading level.
Alignment	Information provided by the vendor about alignment of this instrument to other instruments, standards, etc.	Concurrent validity is reported in the technical manual (Page 56): http://assets.pearsonschool.com/asset_mgr/current/20139/DRA2_Technical_Manual_2012.pdf
Data Reports	Description of data reports that are provided/available at the individual and aggregate level(s).	**Student Reports:**<br>Student Progress Over Time<br>Book Graph (K-3, 4-8)<br>Assessment Summary<br>Continuum<br>Word Analysis and FFI Summary<br>**Class Reports:**<br>Class Completion<br>Class Reporting Form<br>Class Focus for Instruction Summary<br>Class Focus for Instruction Detail<br>Class Word Analysis Group Profile<br>Class Word Analysis Group Profile Detail<br>Class Word Analysis Performance<br>Class Word Analysis Performance Detail<br>Class Word Analysis Task Performance<br>Class Word Analysis History<br>Class Word Analysis FFI Summary<br>Class Word Analysis FFI Detail<br>Historical Reports<br>Class List/Student<br>Students per Reading Level<br>Students per Reading Stage<br>Focus Group

	Demographics<br>Word Analysis Completion<br>Word Analysis Performance<br><br>DRA2 online management system is described here:<br>http://www.pearsonschool.com/index.cfm?locator=PSZw5u&PMDBSUBCATEGORYID=&PMDBSITEID=2781&PMDBSUBSOLUTIONID=&PMDBSOLUTIONID=6724&PMDBSUBJECTAREAID=&PMDBCATEGORYID=3289&PMDbProgramID=23721
Technical Quality	Technical report:<br>http://assets.pearsonschool.com/asset_mgr/current/20139/DRA2_Technical_Manual_2012.pdf

PEARSON CRITERION RELATED VALIDITY ON THE DRA Criterion-related validity refers to the extent to which a measure predicts performance on some other significant measures, (called a criterion) other than the test itself. Criterion validity may be broken down into two components: concurrent and predictive. Concurrent validity correlates the DRA to many other reading tests: Gray's Oral Reading Test-4th Edition GORT-4; Wiederholt & Bryant, 2001 DIBELS Oral Reading Fluency Test-6th Edition; Correlations Between DRA2 and Teacher Ratings

DRA REVIEW, NATALIE RATHVON, PH. D. The following evidence of validation is based upon the review of the DRA completed by: Natalie Rathvon, Ph.D., Assistant Clinical Professor, George Washington University,Washington DC, Private Practice Psychologist and School Consultant, Bethesda, MD (August 2006):

DRA CONTENT VALIDITY. Oral Fluency, running record-derived from only Clay's Observational Survey (Clay, 1993). Teacher surveys (return rates were 46%), conducted (ns of 80 to 175) revealed that DRA provided teachers with information describing reading behaviors and identifying instructional goals.

CONSTRUCT VALIDITY EVIDENCE Results from Louisiana statewide DRA administrations for Spring of 2000 through 2002 for students in Grades 1 through 3 (ns = 4,162 to 74,761) show an increase in DRA levels across grades, as well as changes in DRA level for a matched sample of student (n = 32.739) over a three year period. This indicates that the skills being measured are developmental. The DRA can detect changes in reading levels. As evidenced in two studies evaluating the relationship between Lexile Scale measures and DRA running-record format is a valid method of assessing reading comprehension.

SUMMARY OF WHAT DRA IS: An attractive reading battery modeled after an informal reading inventory based Clay's Observational Survey (Clay, 1993) Instructionally relevant measures of fluency and comprehension. Provides meaningful results for classroom teachers, parents, and other stakeholders. Encouraging evidence that the use of DRA predicts future reading achievement for primary grade students.

DRA CRITERION RELATED VALIDITY: There is a need for studies examining the extent to which individual students obtain identical performance levels on the DRA and validated reading measures.

Student Name: ______________________________________

Kindergarten Emergent Literacy Continuum: Reading and Viewing

Developmental Aspects	Emerging<br>With direct support…	Developing<br>With guided support…	Applying<br>With minimal support…	Extending
**The Child**	With direct support may draw on personal connections to make meaning while participating in a variety of reading/viewing experiences.	With guided support draws on and begins to develop strategies to make meaning (e.g., making connections, predicting, asking questions, and reflecting) while participating in a variety of reading/viewing experiences.	With minimal support draws on, and expands strategies to make meaning (e.g., making connections, predicting, asking questions, and reflecting) while participating in a variety of reading/viewing experiences.	Draws on, expands and begins to identify strategies to make meaning (e.g., making connections, predicting, asking questions, and reflecting) while participating in a variety of reading/viewing experiences.
**Thinking/Metacognition**				
Developing dispositions—awareness, attention, interest, participation, curiosity, engagement, perseverance	With direct support may attend to and may participate in reading/viewing activities (e.g., makes meaning from text using pictures, patterns, memory, prior knowledge).	With guided support engages in reading/viewing activities (e.g., makes meaning from text using pictures, patterns, memory, prior knowledge).	With minimal support purposefully engages in reading/viewing activities (e.g., makes meaning from text using pictures, patterns, memory, prior knowledge).	Purposefully engages in reading/viewing activities (e.g., makes meaning from text using emergent reading strategies).
Setting purposes	With direct support may participate in setting a purpose for reading/viewing.	With guided support sets a purpose for reading/viewing.	With minimal support chooses a purpose for reading/viewing.	Identifies a purpose for reading/viewing; participates in the reading/viewing process.
Processing	With direct support may express some thoughts and understanding before/during and after reading/viewing; may be unrelated to topic.	With guided support expresses some thoughts and understanding before/during and after reading/viewing.	With minimal support expresses thoughts and understanding before/during and after reading/viewing.	Expresses thoughts and understanding before/during and after reading/viewing.
Reflecting	With direct support may participate in the reading/ viewing process; may say something about reading/viewing experience.	With guided support participates in the reading/ viewing process, says something about reading/ viewing experience.	With minimal support participates in the reading/viewing process; reflects on learning—may include purpose, process, experience.	Participates in the reading/viewing process and reflects on learning—may include purpose, process, experience.
**Comprehension/Response**				
Using strategies—use prior knowledge, predict and confirm meaning, ask questions, locate details, create mental images, make inferences	With direct support may use some of the text features (e.g., pictures, patterns, clues from the text) to contribute to discussions before, during, and after reading/viewing; contributions may be unrelated.	With guided support uses some of the text features (e.g., pictures, patterns, clues from the text) to contribute to discussions before, during, and after reading/viewing; contributions are related.	With minimal support uses the text features (e.g., pictures, patterns, clues from the text) to contribute to discussions before, during, and after reading/viewing; contributions are more detailed.	Uses the text features (e.g., pictures, patterns, clues from the text) to contribute to discussions before, during, and after reading/viewing; contributions may include evidence and/or some justification.
Making connections	With direct support may attempt to make a connection to reading/viewing material; connection may seem unrelated to reading/viewing material.	With guided support makes some connection to reading/viewing material.	With minimal support makes connections to reading/viewing material.	Makes connections to simple and complex reading/viewing material.
Retelling	With direct support may retell; retelling may be unrelated to reading/viewing material.	With guided support retelling is related to reading/viewing material.	With minimal support retelling includes some elements of the reading/viewing material (e.g., character, main idea, interesting facts).	Retelling includes elements of the reading/viewing material (e.g., character, main idea, interesting facts); may include the 'gist' of the reading/viewing material.
**Features**				
Demonstrating concepts of print	With direct support may demonstrate concepts of print (e.g., front/back of book, directionality).	With guided support demonstrates some concepts of print (e.g., front/back of book, directionality, points to words on the page).	With minimal support demonstrates many concepts of print (e.g., front/back of book, directionality, points to words on the page).	Demonstrates many concepts of print (e.g., front/back of book, directionality, points to words on the page, tracks with finger using one to one matching).
Recognizing letter-sound relationships	With direct support may be able to name and recognize a few upper or lower case letter-sound relationships.	With guided support is able to name and recognize some upper and/or lower case letter-sound relationships.	With minimal support names and recognizes many upper and lower case letter-sound relationships.	Names and recognizes most upper and lower case letter-sound relationships.
Recognizing words	With direct support may be able to identify a word in the environment (e.g., points to a printed word rather than a picture).	With guided support recognizes a few words (e.g., own name, environmental print, class names, familiar words).	With minimal support recognizes words (e.g., own name, environmental print, class names, familiar words).	Recognizes many words; may begin to use decoding strategies for unfamiliar words (e.g., sight words, environmental print, class names).
**The Support/Scaffolding***	**The Model:** showing, instructing, explaining, directing, making explicit, demonstrating, giving examples	**The Coach:** structuring, sequencing, focusing, cueing, guiding, organizing, supporting	**The Advisor:** suggesting, reminding, prompting, monitoring, asking for elaboration	**The Mentor:** extending, stretching, wondering aloud, exploring, "what if-ing"
***a variety of supports (teachers, peers, environmental, etc.) can be provided at any stage of development**				

Date code: Comments: Final 03/09

Adapted from SD 69 Kindergarten Assessment Committee 2004

Please note: This continuum is not meant to be used as a performance standard

BRITISH COLUMBIA BC Public Service ReadNowBC

Student Name: ______________________________

Kindergarten Emergent Literacy Continuum: Writing and Representing

Developmental aspects	Emerging With direct support…	Developing With guided support…	Applying With minimal support…	Extending
**The Child**	With direct support, may participate in writing/ representing experiences to communicate a message. May understand writing/representing as thoughts written down.	With guided support, participates in writing/ representing experiences by using approximations of emergent symbol systems (a combination of picture, oral description and symbols). Meaning is conveyed more in picture and oral description than in print.	With minimal support, participates in writing/ representing experiences using an emergent symbol system (a combination of picture, oral description and conventional letters) to communicate ideas. Meaning is beginning to be conveyed in the writing.	Participates in writing/representing experiences using a mixture of emergent and conventional symbol systems. Meaning is conveyed in both the writing and the accompanying representations and oral description.
**Thinking/Metacognition**				
Developing dispositions—awareness, attention, interest, participation, curiosity, engagement, perseverance	With direct support may attend to and may participate in writing/representing activities.	With guided support engages in writing/representing activities.	With minimal support purposefully engages in writing/representing activities.	Purposefully engages in writing/representing activities.
Processing	With direct support may voice some thoughts before/ during and after writing/representing; may be unrelated to topic. With direct support may participate in the writing/representing process.	With guided support voices some thoughts before/ during and after writing/representing. With guided support participates in the writing/representing process.	With minimal support voices thoughts before/during and after writing/representing. With minimal support participates in the writing/ representing process.	Voices thoughts before/during and after writing/representing. Participates in the writing/representing process.
Reflecting	With direct support may say something about writing/representing process.	With guided support says something about writing/representing process.	With minimal support reflects on writing/representing process and learning.	Reflects on writing/representing process and learning.
**Purpose**				
Understanding purposes	With direct support may share personal experiences, feelings, ideas, or information in an oral or representational form.	With guided support shares personal experiences, feelings, ideas, or information in an oral/written/ representational form. Beginning to recognize that writing/representing is talk written down.	With minimal support shares personal experiences, feelings, ideas, or information in a written/representational form. Recognizes that writing/representing is talk written down (e.g., mental image matches writing/representing).	Uses writing/representing to share personal experiences, feelings, ideas, or information.
Understanding and using a variety of forms	With direct support may approximate a model to communicate in an oral/representational form (e.g., labels, signs, lists, journals, stories, letters).	With guided support approximates a model to communicate in an oral/representational form (e.g., labels, signs, lists, journals, stories, letters). Beginning to choose a written/representational form for expression of ideas.	With minimal support follows a model to communicate in an oral/representational form (e.g., labels, signs, lists, journals, stories, letters). Beginning to choose a written/representational form that aligns with purpose.	May follow a model or independently select a written/representational form to communicate (e.g., labels, signs, lists, journals, stories, letters).
**Meaning**				
Expressing meaning through drawing	With direct support may draw random scribbles without recognizable forms in a picture.	With guided support attempts to draw a picture that is related to topic and contains some recognizable forms.	With minimal support draws a recognizable picture with some detail that is related to topic.	Draws a detailed picture that is related to topic.
Expressing meaning through print	With direct support may express meaning using representational print forms.	With guided support expresses meaning using representational print forms.	With minimal support expresses meaning using representational print forms.	Expresses meaning using representational print forms.
Explaining writing/representing	With direct support may provide some simple oral detail about writing/representing.	With guided support provides some oral detail about writing/representing.	With minimal support shares ideas or gives information about writing/representing.	Shares ideas or gives more complex information about writing/representing.
**Features**				
Using concepts of print	With direct support may use some concepts of print to represent meaning.	With guided support uses some concepts of print to represent meaning.	With minimal support uses more complex concepts of print to represent meaning.	Uses complex concepts of print to represent meaning.
**The Support/Scaffolding***	**The Model:** showing, instructing, explaining, directing, making explicit, demonstrating, giving examples	**The Coach:** structuring, sequencing, focusing, cueing, guiding, organizing, supporting	**The Advisor:** suggesting, reminding, prompting, monitoring, asking for elaboration	**The Mentor:** extending, stretching, wondering aloud, exploring, "what if-ing"
***a variety of supports (teachers, peers, environmental, etc.) can be provided at any stage of development**				

Date code: Comments:

Final 03/09

Adapted from SD 69 Kindergarten Assessment Committee 2004

Please note: This continuum is not meant to be used as a performance standard

ReadNowBC

Student Name: ____________________________________

Kindergarten Emergent Literacy Continuum: Numeracy

Developmental aspects	Emerging<br>With direct support…	Developing<br>With guided support…	Applying<br>With minimal support…	Extending
**The Child**	With direct support and teacher modeling, may participate in and may attempt to make sense of mathematical experiences.	With guided support, demonstrates a willingness to explore mathematical ideas while participating in problem solving experiences. Is beginning to show an awareness of number, space and time used in everyday life.	With minimal support, demonstrates interest in and willingness to explore mathematical ideas while purposefully participating in problem solving experiences. Communicates an awareness of how number, space, and time are used in everyday life.	Shows interest and curiosity while purposefully exploring mathematical problem solving experiences. Perseveres. Makes and explains connections to number, space and time as used in everyday life.
**Dispositions**				
Developing dispositions—attending, participating; showing interest, curiosity, engagement, perseverance	With direct support, may attend to and may participate in some familiar mathematical problem solving situations.	With guided support, shows interest in and participates in familiar mathematical problem solving situations.	With minimal support, purposefully engages in problem solving, and makes some attempt to solve familiar problem solving situations.	With confidence, curiosity, perseverance uses a range of strategies to make sense of familiar and new situations.
**Processes**				
Communicating using math vocabulary	With direct support, may imitate, copy, repeat a limited math vocabulary.	With guided support, uses and understands basic math vocabulary.	With minimal support, uses and understands a wide math vocabulary.	Uses and understands an extensive math vocabulary including comparative language.
Explaining thinking, using metacognition, and making connections	With direct support, may describe thinking which may or may not be related to the task at hand.	With guided support, is beginning to explain thinking. May need prompts.	With minimal support, makes connections and explains some aspects of thinking.	Explains thinking independently and in detail; makes personal connections.
Representing by building, drawing, or acting out	With direct support, may use materials, pictures, drawings or acting out to represent mathematical ideas.	With guided support, uses materials, pictures, drawings or acting out to represent mathematical ideas.	With minimal support, uses appropriate materials, pictures, drawings or acting out to represent mathematical ideas.	Uses materials, pictures, drawings or acting out to effectively represent mathematical ideas.
**Understanding Shape and Space**				
Matching and sorting	With direct support, sorts and classifies as directed, using an obvious attribute.	With guided support, sorts and classifies using an obvious attribute.	With minimal support, recognizes and describes similarities and differences in order to sort and classify.	Sorts mixed materials on the basis of different attributes; resorts; describes classifications.
Comparing and ordering	With direct support, may compare and order materials on the basis of, e.g., length.	With guided support, compares and orders materials on the basis of, e.g., size.	With minimal support, compares and orders materials on the basis of, e.g., size and shape.	Compares objects, describes differences, orders/sequences, e.g., day plan.
Constructing 3D models of everyday objects	With direct support, uses building materials, and may name the representation.	With guided support, builds and connects the representation to a specific object (e.g., this is a bridge)	With minimal support, builds a somewhat recognizable structure, and describes the representation using simple language.	Builds representations with key features and details, and describes using comparative language.
**Understanding Pattern**				
Identifying, copying, extending, and creating patterns	With direct support, may identify and copy patterns with concrete materials, music, action, and/or language patterns.	With guided support, identifies, copies and extends a given simple repeating pattern, and may create patterns intentionally.	With minimal support, identifies, copies, extends and creates a simple repeating pattern. Beginning to recognize a pattern core or stem.	Identifies, copies, extends and creates patterns of increasing complexity. Describes connections between patterns and recreates patterns in different ways.
Seeing and describing patterns in our world	With direct support, may identify a repeating pattern in our world.	With guided support, identifies a repeating pattern in our world.	With minimal support, identifies and describes a repeating pattern in our world.	Spontaneously identifies and describes repeating patterns in our world.
**Understanding Number**				
Rote counting	With direct support, may join in to a choral count.	With guided support, rote counts with some consistency.	With minimal support, rote counts with consistency.	Rote counts extensively, with fluency and consistency.
Quantifying	With direct support, may count small quantities and may recognize some dot patterns.	With guided support, counts quantities (e.g., to 6 or 7) and recognizes some dot patterns.	With minimal support, counts quantities (e.g., to 10) and recognizes dot patterns, (e.g., dice).	Consistently and accurately counts quantities to 10 (min.) and recognizes number patterns (e.g., dice, ten frames).
Comparing quantities	With direct support, may show which quantity is more or less than another, or the same.	With guided support, matches materials to compare quantities. May use terms more, less, or same.	With minimal support, counts or matches quantities to determine more, less or same.	Recognizes, explains, and models which quantity is more, less, or the same as another.
Matching numerals and sets	With direct support, may recognize/read some numerals and may match numerals and sets.	With guided support, recognizes/reads numerals, and matches numerals and sets with some consistency.	With minimal support, recognizes/reads numerals and matches numerals and sets to 10.	With ease and consistency, works with numerals and sets to 10 and beyond.
Representing numbers	With direct support, may represent number (e.g., by copying the model).	With guided support, represents number (e.g., shows requested number of objects).	With minimal support, uses actions, materials, pictures, words to show how many.	Represents numbers confidently, and in a variety of ways. (e.g., words, pictures, symbols, materials…)
Connecting number to everyday situations	With direct support, may recognize the use of number in everyday situations.	With guided support, connects number to everyday situations. (e.g. birthdays, time, temperature, etc.)	With minimal support, connects number to everyday situations. (e.g. attendance)	Spontaneously connects number to everyday situations.
**The Support/Scaffolding***	**The Model:** showing, instructing, explaining, directing, making explicit, demonstrating, giving examples	**The Coach:** structuring, sequencing, focusing, cueing, guiding, organizing, supporting	**The Advisor:** suggesting, reminding, prompting, monitoring, asking for elaboration	**The Mentor:** extending, stretching, wondering aloud, exploring, “what if-ing”
***a variety of supports (teachers, peers, environmental, etc.) can be provided at any stage of development**				

Date code: Comments:

Please note: This continuum is not meant to be used as a performance standard
