# Supplementary material for: Randomized control trial of Tools of the Mind: Marked benefits to kindergarten children and their teachers
Source: PLoS One. 2019 Sep 17;14(9):e0222447. doi: 10.1371/journal.pone.0222447 (PMC6748407; doi:10.1371/journal.pone.0222447)
Supplement: S3 File — (PDF) [file pone.0222447.s003.pdf]

Items in Blue were asked only in early October.
Items in Green were asked only in May.
Items in Black were asked at both Timepoints.

UBC Kindergarten Teacher Survey

1. Name (Only so I can ask you for clarification later. Your anonymity will be completely protected!)

2. School (Only so I can get background information about your neighborhood should I need it. Neither your name or school will be provided to ANYONE.)

3. Number of years teaching

4. Number of years teaching kindergarten

5. Please describe your classroom

	Choose the number of children
Number of children in your class	
Number of children with special needs	
Number of ESL (English as a second language) children	
Number of children receiving free lunch	

6. In what ways, if any, did the extra $1000 you received this year for supplies help your teaching and/or student outcomes?

7. Do you use aspects of any self-regulation or socio-emotional program in your classroom (e.g., MindUp or Second Step)? What is the name of the program?

8. Did you have children who have difficulty interacting in the classroom?

No	If yes, how many children have difficulty?

9. If you have children who have difficulty interacting: What kinds of negative social interactions do you notice (e.g., defiance toward the teacher or another adult, hitting, fighting, hair pulling, name calling, taking something from another child, refusing to be paired with another child, etc)

10. If you have children who have difficulty interacting: How frequently do these behaviors occur?

Almost never

Occasionally/sometimes 1-2 times a week

Almost every day 3-5 times a week

1-2 times most every day

Several times a day

11. Please indicate your level of agreement with the following statements about most of the children in your classroom

	Agree	Disagree
Overall, children are pretty good about getting back to work after recess		
Overall, children are pretty good about getting right back to work after weekends		

12. Overall, were/are children in your class good about getting back to work after holidays

	(Strongly Disagree)	(Disagree)	(Somewhat Disagree)	(Somewhat Agree)	(Agree)
Overall, they were pretty good about getting right back to work after their Winter break.					
Overall, they were pretty good about getting right back to work after their Spring break.					

13. If someone came in your room, how long did you feel you could talk with that person and let the children in your class work on their own without supervision?

Children could not work on their own at all

1-2 minutes

3-5 minutes

6-8 minutes

9-10 minutes

11-15 minutes

More than 15 minutes

14. Please add any comments you'd like to about your students regarding self-regulation, self-control, or being able to work independently at the beginning of the year or now

15. Classroom Friendships and Community

	Yes	no
In general, do the children in your class show clear preferences for which children they'd like to do things with (e.g., which child they'd like to play with)?		
Do you feel there is/are any child/children who is/are generally less popular in your class and more likely to be left out?		
Have you noticed any cliques forming in your class?		

16. Please indicate your level of agreement with the following questions:

	Strongly Disagree	Disagree	Somewhat Disagree	Somewhat Agree	Agree	Strongly agree
Children in my class are competitive with one another						
Children in my class cheer at one another's successes.						

17. How would you rate the sense of community in your class at beginning of the school year and now?

No sense of community 1	2	3	4	5	6	7	8	9	We're a close knit community 10

18. Please add any comments you'd like to about classroom community, children's acceptance of one another's differences, children's ability to get along with one another, etc.

19. If you have any comments about your children's reading levels or skills please add them here:

20. If you have any comments about your children's oral language, vocabulary, or skills, please add them here:

21. Writing Development

How many of your children were at the following writing levels (independently, without teacher help)? Please count each child only once in your responses and indicate the highest level at which each child was performing (total number should be equal to total number of children in your classroom).

	Number of children
Scribbling	
Drawing a picture	
Can copy their first name	
Can copy sentences from the board	
Write their first name without copying	
Can write most letters when asked to write the letter	
Write initial sounds for many words	
Write simple 2-4 letter words with invented spelling on own	
Write multi-syllabic words with intended spelling with most sounds represented	
Write a full sentence composed by child with invented spelling with most sounds represented	
Write 2 or more consecutive full sentences composed by child with invented spelling with most sounds represented	
Other (please specify)	

22. Writing Development

How many of your children are at the following writing levels (independently, without teacher help)? Please count each child only onceand indicate the highest level at which each child is performing (total number should be equal to total number of children in your classroom).

	Number of children
Scribbling	
Drawing a picture	
Can copy their first name	
Can copy sentences from the board	
Write their first name without copying	
Can write most letters when asked to write the letter	
Write initial sounds for many words	
Write simple 2-4 letter words with invented spelling on own	
Write multi-syllabic words with intended spelling with most sounds represented	
Write a full sentence composed by child with invented spelling with most sounds represented	
Write 2 or more consecutive full sentences composed by child with invented spelling with most sounds represented	
Other (please specify)	

23. If any of your children are writing multiple sentences (I realize everyone might write 0):

	Number of children
How many children can write a string of 2 sentences they compose?	
How many children can write a string of 3 sentences they compose?	
How many children can write a string of 4 or more sentences they compose? (If this a ridiculous option to offer, I apologize)	

24. Are any of your children (I realize everyone might write 0) writing with:

	Number of children
Punctuation	
Capitalization	

25. Please add any comments you'd like to about your children's writing:

26. Please add any comments you'd like about your children's math skills:

27. Your opinions and experiences

At this time in the school year, many teachers start to feel exhausted, burned out, or ready for the summer break. On a scale of 1-10, how would you rate how you are feeling now?

Excited about teaching, Energized	2	3	4	5	6	7	8	9	Exhausted Burned out, weary

28. On a scale of 1-10, how do you feel looking ahead to the next school year?

Excited about starting again, totally energized	9	8	7	6	5	4	3	2	Not looking forward to it, Looking forward to retirement

29. What have you liked/enjoyed most about your class this year?

30. What has been most challenging about your class for you this year?

31. What have you liked/ enjoyed most about teaching this year?

32. What has been most challenging about teaching for you this year?

Thank you again! I am sincerely grateful for your taking time out of your busy day to respond to these questions. *Adele Diamond*

Any other comments are welcome:

Done

Powered by **SurveyMonkey**
Check out our sample surveys and create your own now!
