# Supplementary material for: Randomized control trial of Tools of the Mind: Marked benefits to kindergarten children and their teachers
Source: PLoS One. 2019 Sep 17;14(9):e0222447. doi: 10.1371/journal.pone.0222447 (PMC6748407; doi:10.1371/journal.pone.0222447)
Supplement: S4 File — All Dependent Measures analyzed, with Subsidized lunch, ESL, and Years Teaching as Covariates. (DOCX) [file pone.0222447.s004.docx]

| **S4 – Table**  **All Dependent Measures analyzed,**  **with Subsidized Lunch, English as a Second Language (ESL), and Years Teaching as Covariates** | | | | |  |
| --- | --- | --- | --- | --- | --- |
| **Dependent Variable** | **Analyses controlling for % receiving Subsidized Lunch, centered** | | **Analyses controlling for % ESL, centered** | **Analyses controlling for Years of Teaching, centered** |  |
| Comparing children’s improvement in reading over the kindergarten year in *Tools* classes vs. in Control classes | | | | |  |
| **Improvement in Reading** | χ^2^(1, N = 18) = 4.64, p = 0.02, odds ratio = 3.25 Covariate: p = 0.03 | | χ^2^(1, N = 18) = 4.72, p = 0.02, odds ratio = 3.30 Covariate: p = 0.03 | χ^2^(1, N = 18) = 4.30, p = 0.03, odds ratio = 3.05 Covariate: p = 0.22 |  |
| Comparing the percentage of children who were reading at Grade 1 level or better by May in *Tools* classes vs. in Control classes | | | | |  |
| **% Reading at > Grade 1 level** | F(1,15) = 6.67, p < 0.02, ηp² = 0.33 Covariate: p = 0.13 | | F(1,15) = 6.43, p < 0.02, ηp² = 0.32  Covariate: p = 0.38 | F(1,15) = 4.39, p = 0.05, ηp² = 0.24  Covariate: p = 0.67 |  |
| Comparing the percentage of children who were still non-readers by May in *Tools* classes vs. in Control classes | | | | |  |
| **% Non-readers** | F(1,15) = 6.02, p = 0.02, ηp² = 0.29 Covariate: p = 0.05 | | F(1,15) = 5.31, p < 0.05, ηp² = 0.27  Covariate: p = 0.08 | F(1,15) = 4.76, p < 0.05, ηp² = 0.26  Covariate: p = 0.80 |  |
| In *Tools* classes only, comparing improvement in reading over the kindergarten year by lower-income children vs. those more economically advantaged. | | | | |  |
| **Improvement in Read-ing: Lower vs. Higher SES; *Tools* classes only** | n/a | | χ^2^(1, N = 9) = 4.17, p = 0.12 [NS], odds ratio = 2.05 Covariate: p = 0.63 | χ^2^(1, N = 9) = 4.28, p = 0.11 [NS], odds ratio = 2.11 Covariate: p = 0.26 |  |
| In *Tools* classes only, comparing improvement in reading over the kindergarten year by how far along the children were in reading in September (regression of change in reading level on initial reading level)* | | | | |  |
| **Improvement in Read-ing by Initial Reading Level; *Tools* only** | F(2,6) = 18.18, p < 0.005, R^2^ = 0.89  Covariate: p = 0.72 | | F(2,6) = 11.61, p < 0.01, R^2^ = 0.80  Covariate: p = 0.62 | F(2,6) = 11.64, p < 0.01, R^2^ = 0.80  Covariate: p = 0.38 |  |
| Comparing children’s improvement in writing over the kindergarten year in *Tools* classes vs. in Control classes | | | | |  |
| **Improvement in Writing** | χ^2^(1, N = 18) = 20.20, p < 0.001, odds ratio = 26.18 Covariate: p = 0.25 | | χ^2^(1, N = 18) = 19.90, p < 0.001, odds ratio = 26.01 Covariate: p = 0.32 | χ^2^(1, N = 18) = 19.05, p < 0.001, odds ratio = 25.50 Covariate: p = 0.87 |  |
| Comparing the percentage of children able to write a sentence or consecutive ones they themselves composed with most sounds represented in *Tools* classes vs. in Controls classes | | | | |  |
| **% able to write an original sentence or consecutive ones** | F(1,15) = 18.10, p < 0.001, ηp² = 0.55  Covariate: p = 0.47 | | F(1,15) = 18.24, p < 0.001, ηp² = 0.55  Covariate: p = 0.29 | F(1,15) = 16.43, p < 0.001, ηp² = 0.52  Covariate: p = 0.98 |  |
| Comparing the percentage of children able to write > 1 sentences they composed with most sounds represented in classes taught by the teachers assigned to *Tools* in the year before Tools was implemented vs. Year 1 of *Tools* | | | | |  |
| **% able to write > 1 original sentences the year before *Tools* vs. Yr 1 of *Tools***  **(same teachers both years)** | | χ^2^(1, N = 8) = 13.54, p < 0.01, odds ratio = 9.42 (Data were available for 8 of the 9 Tools teachers because for one *Tools* teacher, Year 1 of *Tools* was her first year teaching. Data on subsidized-lunch and ESL status were not available for the pre-*Tools* year at the class level. Teacher’s years of experience was completely confounded with pre-*Tools* year versus Year 1 of *Tools* in this within-teacher comparison.) | | |  |
| In *Tools* classes only, comparing improvement in writing over the kindergarten year by lower-income children vs. those more economically advantaged. | | | | |  |
| **Improvement in Writ-ing: Lower vs. Higher SES; *Tools* classes only** | n/a | | χ^2^[1, N = 9] = 3.37, p = 0.17 [NS] odds ratio = 1.83 Covariate: p = 0.25 | χ^2^[1, N = 9] = 2.66, p > 0.20 [NS] odds ratio = 1.24 Covariate: p = 0.83 |  |
| Comparing children’s improvement in math over the kindergarten year in *Tools* classes vs. in Control classes | | | | |  |
| **Improvement in Math** | χ^2^(1, N = 18) = 2.50, p = 0.11 [NS], odds ratio = 1.56 Covariate: p = 0.45 | | χ^2^(1, N = 18) = 2.54, p = 0.11 [NS], odds ratio = 1.56 Covariate: p = 0.42 | χ^2^(1, N = 18) = 1.50, p > 0.20 [NS], odds ratio = 1.12 Covariate: p = 0.64 |  |
| Comparing the percentage of children in May who could do no better than count up to 20 objects | | | | |  |
| **% able to do no better than count up to 20 objects** | F(1,15) = 3.16, p = 0.10 [NS], ηp² = 0.17  Covariate: p = 0.39 | | F(1,15) = 2.68, p = 0.12 [NS], ηp² = 0.15  Covariate: p = 0.59 | F(1,15) = 2.62, p = 0.13 [NS], ηp² = 0.03  Covariate: p = 0.49 |  |
| Comparing the percentage of children in May who could do simple subtraction in *Tools* classes vs. in Control classes | | | | |  |
| **% able to do simple subtraction** | F(1,15) = 1.77, p = 0.20 [NS], ηp² = 0.11 Covariate: p = 0.56 | | F(1,15) = 1.94, p = 0.18 [NS], ηp² = 0.11  Covariate: p = 0.47 | F(1,15) = 1.88, p = 0.19 [NS], ηp² = 0.11  Covariate: p = 0.46 |  |
| Comparing the percentage of children in May reported to be having problems interacting with other children in *Tools* vs. Control classes | | | | |  |
| **Problems interacting with other children** | F(1,15) = 6.83, p < 0.02, ηp² = 0.31  Covariate: p = 0.51 | | F(1,15) = 6.37, p = 0.02, ηp² = 0.30  Covariate: p = 0.59 | F(1,15) = 6.06, p = 0.02, ηp²= 0.29  Covariate: p = 0.99 |  |
| Comparing the change from Sept. to May in the percentage of children reported to be having problems interacting with other children in *Tools* vs. Control classes | | | | |  |
| **Change in % having problems interacting with other children** | F(1,15) = 20.59, p < 0.001, partial eta squared = 0.58 Covariate: p=0.007 | | F(1,15) = 15.81, p < 0.001, partial eta squared = 0.51 Covariate: p=0.004 | F(1,15) = 15.13, p < 0.001, partial eta squared = 0.50 Covariate: p=0.06 |  |
| Comparing whether or not the teacher noticed any cliques in May in *Tools* classes vs. in Control classes | | | | |  |
| **Presence of > 1 Clique** | χ^2^(1, N = 18) = 11.99, p < 0.001, odds ratio = 15.77 Covariate: p < 0.001 | | χ^2^(1, N = 18) = 6.48, p < 0.01, odds ratio = 7.72 Covariate: p = 0.35 | χ^2^(1, N = 18) = 6.01, p = 0.01, odds ratio = 7.11 Covariate: p = 0.60 |  |
| Comparing whether or not the teacher noticed any child who tended to be ostracized or left out in *Tools* vs. Control classes in May | | | | |  |
| **Presence of > 1 ostra-cized or left-out child** | χ^2^(1, N = 18) = 4.87, p = 0.02, odds ratio = 3.45 Covariate: p = 0.53 | | χ^2^(1, N = 18) = 6.63, p < 0.01, odds ratio = 8.30 Covariate: p = 0.53 | χ^2^(1, N = 18) = 3.21, p = 0.07 [NS], odds ratio = 2.2 Covariate: p = 0.64 |  |
| Comparing whether or not the teacher reported students were good at getting back to work after recess and weekends in *Tools* classes vs. in Control classes in May | | | | |  |
| **Getting back to work after recess and weekends** | χ^2^(1, N = 18) = 5.31, p < 0.02, odds ratio = 5.28 Covariate: p = 0.46 | | χ^2^(1, N = 18) = 5.04, p = 0.02, odds ratio = 5.04 Covariate: p = 0.42 | χ^2^(1, N = 18) = 6.69, p < 0.01, odds ratio = 8.39 Covariate: p = 0.13 |  |
| Comparing whether the teacher reported students had been good at getting back to work after Spring break in *Tools* vs. Control classes | | | | |  |
| **Ability to get back to work after Spr. break** | χ^2^(1, N = 18) = 4.92, p = 0.02, odds ratio = 3.50 Covariate: p = 0.13 | | χ^2^(1, N = 18) = 4.33, p < 0.03, odds ratio = 3.05 Covariate: p = 0.61 | χ^2^(1, N = 18) = 3.81, p = 0.05, odds ratio = 2.6 Covariate: p = 0.90 |  |
| Comparing # of minutes teachers reported their students could be left to work on their own, unsupervised, in *Tools* vs. Control classes in May | | | | |  |
| **# of minutes could work unsupervised** | F(1,15) = 11.43, p < 0.005, ηp² = 0.43  Covariate: p = 0.76 | | F(1,15) = 14.98, p < 0.005, ηp² = 0.50  Covariate: p = 0.04 | F(1,15) = 12.96, p < 0.005, ηp² = 0.46  Covariate: p=0.32 |  |
| Comparing *Tools* and control teachers’ excitement about teaching in May. (Because the distributions were so skewed, we compared the % endorsing choices 1 or 2 (excited about teaching, energized) to the % endorsing any other choice on the 10-point scale.) | | | | |  |
| **Teachers’ excitement about teaching in May** | χ^2^(1, N = 18) = 4.99, p = 0.02, odds ratio = 3.58 Covariate: p = 0.71 | | χ^2^(1, N = 18) = 4.29, p < 0.03, odds ratio = 3.00 Covariate: p = 0.27 | χ^2^(1, N = 18) = 4.26, p < 0.03, odds ratio = 3.00 Covariate: p = 0.37 |  |
| Comparing *Tools* and control teachers’ enthusiasm in looking forward to the next school year. (Because the distributions were so skewed, we compared the % endorsing choices 1 or 2 (extremely enthused) to the % endorsing any other choice on the 10-point scale.) | | | | |  |
| **Teachers’ enthusi-asm looking forward to the next school yr** | χ^2^(1, N = 18) = 5.67, p < 0.02, odds ratio = 5.86 Covariate: p = 0.73 | | χ^2^(1, N = 18) = 7.71, p < 0.01, odds ratio = 10.86 Covariate: p = 0.25 | χ^2^(1, N = 18) = 5.67, p < 0.02, odds ratio = 5.86 Covariate: p = 0.02 |  |

* We would have done a similar analysis for writing and for math but there was too little variation between children in the Fall levels of writing or math competence.

Gray font indicates non-significant results.

ηp² = partial eta squared

χ^2^ indicates a generalized estimating equation analysis was used, from which a chi square was generated.
