# Supplementary material for: Randomized control trial of Tools of the Mind: Marked benefits to kindergarten children and their teachers
Source: PLoS One. 2019 Sep 17;14(9):e0222447. doi: 10.1371/journal.pone.0222447 (PMC6748407; doi:10.1371/journal.pone.0222447)
Supplement: S5 File — (PDF) [file pone.0222447.s005.pdf]

S5 - Comments by Teachers, Parents, and Principals

The topics covered here are:

- **General**
- **Reading**
- **Vocabulary and Oral Language**
- **Writing**
- **Math**
- **Getting Along Together; Lack of Fighting and Social Exclusion**
- **Children Helping and Supporting One Another**
- **Sense of Community in the Classroom**
- **Ability to Work Independently**
- **Self-Regulation / Attention Regulation**
- **Joy in Learning and Enjoyment of School**
- **Teachers' Feelings about Teaching**

General Comments

Tools teachers

"I see the positive outcomes for my students in all aspects of their learning! This really is making a difference!"

Control-group teachers

Parents

Parent #1: "I cannot speak highly enough of the Tools of the Mind program. My son has developed and matured so profoundly since the beginning of the school year that is difficult to summarize in a few sentences.

I have watched him become excited and continually interested about learning everything. He began the year with little interest in reading or imaginative play. Now he tells his father and I a chapter in his 'story' (a book he is writing in his head) every night. He guides his 3 year old sister and friends outside of school in imaginative play and storytelling. He wants to read chapter books and is determined to finish reading/hearing the Treehouse series of books. He has come home and asked to do 'homework', taking time each day to practice his skills by doing mazes, coloring, working on letters or trying math. All this is self-directed. At the beginning of the year it was difficult to even get him to sit for 5 minutes to color a page and now he readily takes responsibility for himself and his actions.

This program has gone a long way towards instilling my child with exceptional abilities that will take him through life inside and outside of school. As a mom I have the best intentions of working on my children's learning outside of school but in this busy ol' world reality and intention don't always work together. I have found that the way my son is taking control of himself has made it much easier to create support for him at home. It discourages helicopter parenting in the best possible way. At 5 years old I see skills growing in him that I sadly find missing in people 15 or more years his senior. This is a wonderful program and the effects have been profound and astounding in our lives. I sincerely hope that, when the time comes, I will be able to have my daughter in the Tools of the Mind program so that she will have the same significant start in school and learning as my son."

Parent #2: "Below are some thoughts on Tools of the Mind. How do you sum up such a great program in a few words??? I am writing as a parent of a Kindergarten student who is part of the Tools of the Mind Program. I have also had the opportunity to volunteer regularly in the classroom and observe the progress that the children are making. Without exception, the children in the class have enthusiastically embraced each of the different themes presented and have eagerly anticipated each new book being introduced. Their enthusiasm for the materials has carried over to their play centres in the classroom where they have used the themes effectively in their free play.

I have observed students incorporating writing into their play centres through the use of white boards and notebooks. Writing is not a chore for them but something that they embrace and incorporate as part of their free play. They also use the themes for dramatic play outside at recess. All of this is student-led with roles and characters being discussed and negotiated as they leave the classroom on their way outside. Their written and verbal literacy skills have improved a great deal since the beginning of the year but so has their ability to negotiate and find solutions to problems without the need for an adult to assist. They are able to recognize their differences, accept them and find a solution where required.

In terms of my own child's progress, it is wonderful to see how he embraces learning and looks forward to being in the class each day. Learning to read has been a fast and painless process as he is able to sound out letters and figure word sounds out on his own for the most part. It has required very little parental input and it is amazing to see him reading full books when he was just beginning to sound out three letter words at the beginning of the year. His written sentences are legible, appropriate to the context and his oral story telling abilities are astounding."

Parent #3: "My child has had the privilege of attending a Tools of the Mind Kindergarten class this year. As a parent and educator, I have observed such wonderful social, emotional and intellectual growth within my child's development. As a parent, I have observed [my child's]:

* **willingness to take risks and try new things** as a learner flourish
* increasing **ability to focus** for longer periods of time on more challenging tasks
* **excitement for reading** grow. She not only loves listening to stories, but has recently been bitten by the "reading bug." [My child] will spend her free time independently reading simple patterned stories without any encouragement from adults.
* **confidence grow as a developing writer.** She confidently prints letter sounds, draws detailed pictures and enthusiastically shares her stories with her family. She considers herself a writer.
* **enthusiasm towards school.** She always has a story to share about her day and is always excited about going to school.
* **sense of belonging and relationships grow between her and her classmates.** [My child] often talks about the children she has worked with within her group and has become acquainted with **all** of the children within her class. She often shares stories about a variety of children she plays with at school.

Above all, I have noticed [my child's] excitement for learning and her inquisitive nature continue to develop. As a family, we all love listening to her share her stories "Mommy, Daddy, and Megan did you know that…"

My older son had the same teacher last year before the Tools of the Mind program was introduced. I sometimes find it hard to believe it is the same classroom with the same teacher as the entire feel of the class has changed. She is still the same amazing teacher as before but the students have so much more self control. They take an active role in the classroom and in their learning and are able to self regulate to a degree that adult intervention is rarely required."

Parent #4: "My daughter rushes out of school full of excitement about Jack and Annie [characters in the storybooks they have been reading], what they're doing, what will happen next, and she details for me all that she's learning [in Tools of the Mind].

Right up until Spring Break, the children were regularly playing "Jack and Annie" outside the classroom; at lunch, after school, on playdates. I also think they connected socially on a different level because of the activities in the classroom. She's happily playing with kids that she wouldn't have played with last year, and their play feels free

to me. Their play is wonderfully creative. In the fall, many of the girls (and some of the boys) engaged in an ongoing imaginative game about “turtle island” where they used the sandy playground to draw out a hotel (“turtle hotel”) with rooms for each, and a track (for [my daughter] to run), and a kitchen. The game went on for weeks and was more creative and generative than I’ve really ever seen on the playground before.

The integration of fictional characters with factual information has really expanded her range of interests. She pursues additional knowledge in areas that really pique her interest. She brought home library books about the rainforest and animals you would find there when they were doing the rainforest book [remember she is in Kindergarten!], and she continuously makes connections between what we might be doing in our day and what she’s learned at school. Our family really enjoys fiction, so I like seeing that her comfort with different genres of books has also increased.

The quality of the children’s art is surprising in that all children are really producing amazing work—detailed and bright—perhaps because much of the art is linked to the learning that excites them. My daughter not only wanted me to admire the mummy’s mask she made, but she wanted to explain to me what it was and all the death rituals of ancient Egyptians. The effort and time she and a friend put into a dragon (for Chinese New Year) and on figuring out how to draw a horse for a farm was inspiring. They started the work in school, but brought it home to finish—again, so excited and inspired by what they are doing and exposed too.

I also credit the program with encouraging her to challenge herself. She’s clearly inspired by what she can find in books and happily picks up books beyond her year level. What’s interesting to watch is that she is applying strategies she’s learned, and it’s expanding her capacity. It’s not that I feel any desire for her to be reading above grade-level. The point is that she sees learning as something accessible to her. She’s excited to learn and doesn’t identify barriers; she just tries to overcome them.

This program has made me realize that our standards, or expectations, for what children can achieve are limiting and restrictive. In an environment that creates excitement AND skill development, children willingly investigate concepts and learn. My daughter has at least. In my opinion, she is exploring knowledge for its own sake, uncovering and engaging with ideas, and enjoying herself. Yes, she’s working, but it’s so joyful that I just wish she could keep going in this kind of approach throughout her K-3 schooling.”

Principals

“[The Tools teacher in my school] is a very experienced, extremely talented teacher. For her to say Tools is making a difference for kids is quite something because everything she does makes a difference. She has some new “tools” and is telling me she is getting better results than ever before.”

The 2 Coaches of *Tools of the Mind* teachers

“Working with the Tools of the Mind teachers has been a joy. It is amazing to see teachers who were hesitant at first to take on an extensive program gain momentum the more they learned about Tools and the more they saw results in their classrooms. Teachers tell us over and over that they are amazed at what their children have accomplished and how the program is so seamlessly interconnected in math, science, art and language. It creates a learning context familiar to the children where they can explore academics rather than receive knowledge from the top down. As the year has progressed, teacher’s enthusiasm has grown with their knowledge of the program and the capability of the children.”

Comments on READING

(A few comments are partially repeated under Writing, as they apply to both.)

Tools teachers

“The literacy level in the classroom this year is much higher [than in past years]. We are a new Early Intervention

school so our resource teacher evaluates all the Kindergarten children. In January, no one in my classroom was at risk. That has never happened before. Children who had qualified for ELL [English Language Learner] support at the beginning of the year, no longer qualified in January. That has never happened before. In past years some children were always at risk."

"I have never had a whole class that was reading by May until I did the Tools program. Students are reading many sight words and are able to use all of the strategies for reading that we practice everyday.

"Students are not only able to read (for the most part) but they enjoy it and WANT to do it!!! They also feel such a great sense of pride being able to do it."

"Only 6 children in my class are NOT reading this year. In past years I was lucky to have 6 kids who *were* reading. As of Feb, I had 17/22 students reading at a DRA Level 3, including ELL students – exceeding expectations. As of April, the majority of my students are reading at a DRA Level 6 or above, fully meeting criteria for the first term of Grade 1."

"Normally I only get to A level books with most students (possibly a few to B) but this year I have students reading A-C levels so far."

"Much higher levels of reading and writing for every child [than in past years]. Opportunities for those children who come to school with knowing how to read and write to continue to grow."

"Starting to read in kindergarten is so amazing!!"

"Much higher levels of reading and writing for every child. Opportunities for those children who come to school knowing how to read and write to continue to grow."

Control-group teachers

"The majority of my class know all their letters and sounds. Some are beginning to sound out words. I have four students who are able to read some sight words. I have four students who do not yet know their letters and sounds."

"This year I observe that there are more readers in the classroom than in past years. A lot of work has been done in the area of literacy development. Reading is one of the school goals and this year is the first year our school has received early intervention funding/support."

"Most children can recognize many word families and some sight words, though they can't read a book. I also directly teach phonemic awareness, and administer the ELPATS (a phonemic awareness assessment) and about 80% are not at risk."

Parents of children in *Tools of the Mind*

"Since the beginning of the school year I have noticed a huge change in my son's confidence in reading words. He also writes sentences at home and can explain concepts."

"This is my first experience with a child in Kindergarden so I was not sure what to expect for [our second child] this year. I have been so impressed with what he has achieved. He can sound out and recognize words with increasing frequency; I didn't expect him to be so close to reading at this stage."

"Learning to read has been a fast and painless process as he is able to sound out letters and figure word sounds out on his own for the most part. It has required very little parental input and it is amazing to see him reading full books when he was just beginning to sound out three letter words at the beginning of the year."

"I credit the program with encouraging her to challenge herself. She's clearly inspired by what she can find in books and happily picks up books beyond her year level. What's interesting to watch is that she is applying strategies she's learned, and it's expanding her capacity. It's not that I feel any desire for her to be reading above grade level. The point is that she sees learning as something accessible to her. She's excited to learn and doesn't identify barriers; she just tries to overcome them."

Coach of *Tools of the Mind* teachers

"This method of teaching writing has enabled the children to understand how words and sounds function and they have naturally moved into reading. With the emphasis on helping children learn to write, we see many more children able to read and write….The program accommodates students of all levels so they are stretched whatever their ability."

One of the two creators of *Tools of the Mind*

"It is really, really exciting that we got high literacy scores without pushing but 'following the children's lead' so that children were taught skills when we knew they were ready for them. This shows that teaching reading in a developmentally appropriate way that is responsive to the children can get the same or better results. At no point were children forced to read – and Tools teachers never did phonics drills. I think it is important that these literacy gains are <u>*completely*</u> without teacher-led drills on letters or sounds."

Comments on VOCABULARY and ORAL LANGUAGE

Tools teachers

"All the students are speaking with far richer vocabulary with each other now than at the beginning of the year.

"It is amazing to see how much oral language is being used on a daily basis. They are quick to experiment with new vocabulary. They love to talk and interact with each other as they play, as they eat, and as they work."

"Children use rich, theme-related vocabulary in proper context. They also extend this language out on the playground, and in other discussions. They make many connections with various texts and real world situations."

"The language in our classroom is very rich! They love having discussions about the topics we are learning about and the students are so excited about the topics that they go home and do even more research. Plus students are always surprising me with connections they have made between topics and books we have already read in class.

"There is lots of conversation which is on topic and connected to our themes."

"I see more conversations and negotiating with each other."

"Children use oral language skills to solve their problems. They use the vocabulary in their dramatization. And it is amazing to see how much oral language is being used on a daily basis. They are quick to experiment with new vocabulary. They love to talk and interact with each other as a play, as they eat, and as they work."

"They are able to articulate what they are working on and know how to get there."

Control-group teachers

"Their phonemic skills have increased immensely, and their oral language has increased as well (especially in social play). I mostly notice them using vocabulary from the science texts we read."

"Most of my students have excellent verbal skills."

Comments on WRITING

(A few comments are partially provided above under Reading, as they apply to both.)

Tools teachers

"The writing my students produce is personal and meaningful. Even days later they can re-tell what they have written. This had never occurred in any of my kindergarten classes before….The children's writing is constantly

improving and they strive to write more. They want to write, they want to be heard, and they transfer this skill set in to other areas of their lives."

"Writing growth is profound. My ESL resource teacher has never seen such growth of Kindergarten students in her entire teaching career. (She is close to retirement.) Every child is excited to write – even the weaker students who are happy to ask for help."

"In my classroom 20/22 children are able to write at least beginning sounds to represent what they have written and are able to remember and re-read what they have written even days later. In previous years, only a few of my students could write a message and be able to re-read what they had written even on the same day."

"Writing has come so far in the majority of students. They are not restrained by a frame or inability to write a word, they can get a message in their mind, remember it, write lines to represent and add letters/sounds. They can write what they want and they can (with big vocabulary words and detail) – it is empowering for them!... This is the first time in my 6 years as a primary teacher that 17/20 of my students are meeting or exceeding grade level expectations for writing. I have students writing and sustaining focus… the program has really helped learners who would have struggled much more."

"Amazing writing development. Some kindergarten children are writing up to 3 sentences (some even more) which is very exciting. My students are now confident writers."

"Writing like I've never seen before! I have 2/3 of my class writing meaningful sentences – 1/3 of those are actually writing multiple sentences. The remaining 1/3, who are not yet writing sentences, understand the process of writing and are beginning to fill their lines with initial and end sounds."

"The literacy level in the classroom this year is much higher. We are a new Early Intervention school …. This year I find that every child in my class can write a sentence by themselves. More children than ever before are able to write more than expected. It has been very rewarding and exciting to see. It is also exciting to read chapter books to the students. The topics in the books really make learning exciting for the students. There is rarely anyone who complains that they don't know what to write. Compared to previous years in which students had a lot of trouble thinking of ideas to write and it was like pulling teeth to get them excited for writing time. <u>At this time of the year, I have never [in 20 years of teaching] seen such growth in writing nor as many students exceeding writing expectations</u>. Never dreamt I`d see kindergarten children writing full sentences, much less most of the children doing so."

"This year I have a greater % of students writing and <u>wanting to</u> write. Their output is meaningful and it shows them using their "tools" as a writer and a learner. This of course has transferred into an ability for and desire to read – I am so confident in the students I am sending to Grade one! ☺"

"Much higher levels of reading and writing for every child [than in past years]. Opportunities for those children who come to school knowing how to read and write to continue to grow. Even the lowest child who is a beginner ELL student has shown growth in being able to formulate a message that has something to do with what we have been reading and a picture to go with his writing."

"I am extremely impressed with all of our kids' ability to write. We are an inner-city school with many (almost half) at-risk. Our lowest-performing child (who is awaiting a Ministry designation) who was unable to orally put a sentence together was able to tell me yesterday that "the" is spelled "T-H-E". This is HUGE!"

"The students' writing and reading is amazing! An LST [Learning Support Teacher] came to look at one of my lower students' written output. When she saw his writing she wondered why I had concerns. So I showed her the work from other students, including my ELL students and an English language learner with speech issues; she was amazed at how well and fast the entire class was progressing and quickly realized why I had concerns."

"I have enjoyed seeing the enormous progress my students have made in writing and reading. I have never had so many students writing 2 or 3 sentences by the end of Kindergarten. And all of my students are able to write at least one sentence independently with most of the sounds."

Control-group teachers (only one commented on writing)

"Writing has been a focus area and I observe a higher number of children engaging in some form of writing independently."

Resource teacher

"I am amazed at the quality and level of writing in the Tools kindergarten class I have been servicing. The sound maps are amazing!!! Because I service all 3 kindergarten classes in our school I am well aware of the differences in each class in terms of writing."

Parent of a child in *Tools of the Mind*

"Writing is not a chore for them but something that they embrace and incorporate as part of their free play."

Principals

"The writing that comes out of the Kindergarteners in Tools is amazing."

"I have noticed in our Tools class that all the children are so focused on their writing during journal time. They are very 'engaged' in their writing."

The 2 coaches of *Tools of the Mind* teachers

"We have observed that "Scaffolded Writing," the unique way writing is taught to the kindergarten children in the Tools classrooms, is having a very positive effect on their progress in this area. Most children were not writing in September but by May all but a few are. There are a number who can write two to three sentences on their own using complex sentence structure, sophisticated vocabulary and conventional spelling. There are only a handful of students in each class who are at the beginning stages of writing only the beginning sounds. These results are consistent across all socio-economic areas. We have never seen such advanced writing in Kindergarten before."

Comments on MATH

Tools teachers

"Students this year understand the concepts behind the math. It has given them a solid foundation to build upon."

"Students have asked to play math games from class at home."

"The games are engaging and the students are able to 'play' independently and they are developing key early numeracy skills. There are so many activities interwoven through the days in a variety of ways. My students love the various pattern activities with sounds and movements."

"Children are counting with increased confidence forwards and backwards."

"I'm not sure if the math is any different as a result of this program."

Control-group teachers

"I really appreciated the proD from the summer--I have used the concepts I learned in the math workshop throughout the year and it has helped immensely."

"I feel this year I have really improved in my numeracy teaching (thank you for the Math ProD workshop) and the children have a stronger number sense than in previous years."

Comments re: GETTING ALONG TOGETHER; LACK OF FIGHTING and SOCIAL EXCLUSION

Tools teachers

"There have been less issues that have come to my attention from the lunch supervisors [this year] because my students have a plan before they go out to the play. I will often hear them say things like, 'Let's play hospital. I'm the ambulance driver. You can be the sick person.' My students seem to be much better at negotiating with each other."

"The students are speaking to each other more, independently able to work through disagreements and solve problems with their peers through compromise and negotiation….They are willing to work and help any peer in the classroom. There are able to solve disagreements quite independently and there is way less tattling behaviours that used to take up a great deal of class time after recess and lunch breaks."

"I notice of course the children's preferences for friends to play with and sometimes small conflicts arise. However, this year I have been very impressed with students' abilities to work with everyone in their different groupings and I notice how supportive they are of each other."

"More willingness to interact with anyone this year. Every student is willing to work/play with any peer in the classroom vs. previous years when there were 'popular' vs. 'unpopular' problems and kids who didn't fit in."

"I have NO refusals to work with each other (regardless of ability, gender, age, culture, special needs). That would have been unheard of in past years."

"No one makes faces or puts up a fight when I partner students together. They seem more accepting of working with everyone in the classroom [than in past years]."

"Students' understanding and practice of social 'rules' is much improved. They take Tools into free play. I have had students ask me if they can remove themselves from the classroom to discuss their problem and come to a solution. Then they come back to me and tell me that they have fixed the problem. This is amazing!"

"I love hearing the children using the dispute bag to figure out who goes first and many of the skills that have been taught. They independently use these skills and don't often have to be reminded by the teacher. They are quick to share with others, those who may be new to our classroom, on the rules and expectations of our classroom. They 'help' the visitors follow our classroom rules. One of the class' favorite themes was the theme of Ninjas. The other kindergarten teacher asked me if my children were fighting outside on the playground at recess because of the ninjas. I said no. The children knew that the ninjas were in control of their bodies and that's what they wanted to do as well."

"I love the positive social interactions I see between the students. Students are able to work together with peers more effectively; there are less conflicts within the classroom between students. The students are willing to try new centres and choose to work with a variety of peers during free play."

"Students are more willing to work out disagreements and make compromises as well as help peers who need help. Students are willing to share their feelings more openly in a group setting and work together to find solutions and willing to revisit if it doesn't work and try something new. They are able to negotiate tasks and do it fairly."

Control-group teachers

"I still have children [in May] who have difficulty interacting. The kinds of negative interactions I see are: Mean statements -withholding of items or information (not sharing) - hitting, grabbing, pushing - name calling - bossy - not including others in play - refusal to be paired up with a child - taking something from someone else - defiance towards the teacher or another child - teasing - running away from a peer who wants to join in the play - laughing at another's expense - purposely bothering another (i.e. rubbing their head, taking their shoe away)."

"At this point [early May], social blackmailing continues to be an issue, as well as hitting (between the same girls who have social difficulties)."

"I find that since we have come back from spring break my students behaviour has regressed. At the beginning

of the year they didn't know 'the rules', and now they seem to have forgotten all about them again!! I am having to constantly monitor behaviour and 'put out fires'. Maybe I'm just too tired and its affecting my perspective on things!... I think that it is ridiculous that I have to send my kindergarten students out on a poorly supervised playground with 400 other students. I feel that this has resulted in increased behaviour issues both in and out of the classroom."

"Sadly, although there have been improvements, I would have to say I still have 9 children who are having difficulty interacting (e.g., refusal to share, tantrums). The physical aspects towards others have been reduced (e.g., less hitting, slapping, kicking, stealing, throwing furniture, breaking classroom supplies, hair pulling, etc.)"

"At the beginning of the year, the students needed a lot of help problem solving, and playing with more than one friend. Now students are needing less prompting when expressing their feelings to friends. They still need lots of help to negotiate play."

"The classroom is quite diverse….4 children have great difficulty self-regulating and controlling their actions/impulses and or behaviour."

"I would stay that many more than 5 of my students who have difficulty interacting - defiance towards adults, physical agression towards adults, fighting (both physically and verbally), name calling, taking something from other children, taking things from the teacher/classroom, refusing to be paired with another child either for work or play, and many other negative behaviours….Although as I mentioned, I have many students with behaviour challenges, I have many well-adjusted, thoughtful and ready to learn students in my class this year."

"At this time in the year [May] many students need mediated support to be respectful of one another's differences, to include others in their play, to advocate for their needs and to respect their peers' needs."

"Still see some defiance toward teacher and SSW, and hitting of other children."

"I am very interested in helping students to master their ability to self-regulate their thoughts, actions and behaviours and in supporting them to become kind, generous, and considerate people who have an awareness of their needs and the needs of others. I am also very interested in how to help students to realize their strengths and to use their strengths to work on areas of challenge in both themselves and others. The pace of my Kindergarteners' day is VERY fast and I would love to slow this down and give more time to self-reflection, contemplation, drawing, singing, and experiencing beauty and wonder and enjoyment of the outdoors. I am interested in setting up my classroom and programming in a proactive and thoughtful way that honours children's need to play and their need to learn how to 'be' within the context of a group."

Comments re: CHILDREN HELPING AND SUPPORTING ONE ANOTHER

Tools teachers

"The students in my class get along with each other. They may have a preference for a child they would like to play with. However, it is usually because the child is interested in the same activity. Boys and girls play together, boys play with boys and girls play with girls. It is a mixed but close community. I am told about a child being hurt on the playground by a number of children. In years past, they have not helped each other to this degree, when a child was hurt but now have witnessed many students going to another students' aid. I see our classroom has a warm and accepting place. One mother, the mother of the student with extreme anxiety, went to the principal near the winter holiday time to tell the principal how much her daughter loved her teacher. The mother then came to me saying how thankful she was, as every day in China, her daughter did not want to go to school and now wanted to go to school (even when sick). The mother was extremely happy that her child felt safe and loved in our community."

"They offer help and assistance when needed without being asked and without belittling the struggling student. They look out for one another and ensure everyone has someone to play with or talk to….This behaviour even spills out to the outside playground."

Everyone plays and learns with all students. All of my students celebrate the efforts and successes of each child – regardless of ability. They also offer help and assistance when need without being asked or without belittling the struggling student. They look out for one another and ensure everyone has someone to play with or talk to… This

behaviour even spills out to the outside playground – it is truly AMAZING!

“I think the majority of students in my class are able to get alone with one another very well. They love to help and support each other and they are very kind and considerate to each other's feelings. The Tools program has really helped them feel comfortable working with other students in the class because they know that they will be working with different students each week. I also think the way the Tools program incorporates roles and responsibilities helps the students to accept their role or job in their group and there is no arguing over who has to do what job.”

Students work willingly to help their peers during our day. When a new student joined our class with severe behavioural issues they were very accepting and tried to help this student integrate into our classroom routines.

They are much better at helping each other and they take their role as a buddy checker seriously. They like to make sure their buddy completed their work.

“Socially I have noticed that the students this year are more comfortable working with other students in the classroom.

“My students this year are very inclusive and are able to work with anyone in the classroom. Strong bonds between individual children and between all children. Children who may not have previously played with each other do.”

“Students are now able to support each other without teacher involvement which is different from previous years.”

“Students not comparing themselves to each other academically. They are cheering each other’s success, are more supportive of each other.”

Control-group teachers

“They tend to get along pretty well at this point in the year; we have some strong leaders who are 'friends with everyone' who, when they are present, are a very positive influence (will remind about appropriate social skills and behavior, ‘you need to apologize for that’, ‘we don't do that here’, etc). We also have a few children who have a very difficult time acting kind most of the time. This makes it difficult to have a totally close knit community, as these children, while they have progressed, still need significant support to make choices that benefit everyone and not just themselves.”

“At this time in the year many students need mediated support to be respectful of one another's differences, to include other's in their play to advocate for their needs and to respect their peer's needs.”

“Most challenging this year has been the lack of an established and harmonious classroom community where kindness, consideration and care are the norm….The students are learning to read and write, but their ability to be well-adjusted and considerate human being lags behind.”

Comments re: SENSE OF COMMUNITY IN THE CLASSROOM

Tools teachers

“My students this year have a strong sense of community – in fact we are a strong-knit FAMILY. Everyone works, plays, and helps EVERYONE – without any moaning and groaning. They are far more adaptable, flexible and accepting of everyone regardless of appearance or ability.”

“More of a sense of community [this year]. I see children helping each other and looking after each other to a greater degree from in the classroom to out on the playground at recess [than in past years].”

“We have a very strong sense of community. Students are quick to check on each other, if one is crying or angry, and show concern for a peer who is hurt. This I see more often than not.”

"They have a strong sense of community *sense of community.*

"Student peer relationships are fantastic – know, play, and work with EVERYONE. Real sense of community/family."

"We are a tight-knit FAMILY. Have enjoyed seeing the kids working together so that all are successful."

"Last 2 years have been very trying with the student's social/emotional learning. There was very little self-regulation. This year with Tools there has been tremendous growth. They are better able to self-regulate their behaviour and be more patient, kind and inclusive."

Control-group teachers

"Their ability to work cohesively as a community or as a team is inconsistent from day to day and there are many days [even now in May] where the children's energy is 'scattered' and they seem to march to the beat of their own drum."

"Building a strong sense of community has been a challenge this year. At this time in the year [May]…their ability to work cohesively as a community or as a team is inconsistent from day to day."

Comments on ABILITY TO WORK INDEPENDENTLY

Tools teachers

"I see a big difference in the students now in May compared to September. All the students know the routines and can get to work without much prompting. They know the schedule and can start on the activities without my support."

"Greatest reward is…seeing kids so very proud of what they can do independently."

"Peers help their study buddy remain on task and regulate their behavior in small groups, and they require minimal teacher assistance to solve the minor issues that sometimes arise."

"Greatest reward from using Tools this year is children having more control of their own learning."

"Students at this time of year [May] are much more independent than in past years. They are able to look at the chart, find which group they are in and go to that area without any teacher support. They support each other and use peer regulation."

"Students are now able to support each other without teacher involvement which is different from previous years."

"They are able to articulate what they are working on and know how to get there (practice, help from a study buddy, teacher assistance)."

"I'm so thrilled to have children who can work independently! Many to all of my students are now able to work independently. Parents love the independence of the students."

"One way I've changed the way I've managed my classroom is by empowering my students through planning and activities to manage themselves."

"Children can be independent learners even in kindergarten!!"

Control-group teachers

"This is a very young class (most turned 5 in Oct/Nov/Dec) and in the beginning it was very, very challenging. They are so much better now, BUT we have a strong routine, review expectations frequently....I do feel that many have increased in their self-regulation abilities, but I do still need to play an active role in prompting/ modeling/ affirming behavior. They did not come to school with a great deal of independence, and some still need support in managing belongings, time, and behaviour."

The classroom is quite diverse. There are approximately 5 children who are still unable to work independently.

"At the beginning of the year only a few children were able to work independently. Now only a few ask for help continually without first trying on their own."

"I am continuing to struggle with the children listening to all the instructions when given an activity and then follow the instructions independently. I often have 4-5 children who will ask me what they need to do."

"A major challenge this year has been the range in abilities and motivation in my class."

Comments on SELF-REGULATION / ATTENTION REGULATION

Tools teachers

"In the fall I was really struck by the primal sense of the children – random, impulsive, distractible, emotional, the limitations were tangible. There is no comparison to their behaviour now – the beauty being it is second nature….They still are spontaneous but it's more 'appropriate' and not as 'off the wall'. They get tired and lose focus but it is after thinking hard. In my experience free time tended to be a bit crazy and hard to manage. Here it is not."

"The return to school after Christmas and Spring Break was smooth. Usually each return is like a mini-September – poor self-regulation and adjustment to school. Not this year. It was like the children were returning from a weekend away. In fact, on Mondays, return-to-school has also been much smoother. Very little re-adjustment after a weekend away."

"In 20 years I have never been able to come back from school holidays so seamlessly, with minimal learning lags and still have such great retention of information and routine!"

"We had a child move from very, very little regulation to now being unable to distinguish from peers. This child was very dangerous to others in Sept/Oct."

"At the beginning of the year, my students' ability to self-regulate was very limited. Now they are extremely independent, in-control and able to monitor and regulate themselves.

"Seems like there is more on-task behaviour and when students are off-task they are able to return to tasks easier."

"Many to all of my students are able to work independently. If there changes in the schedule or a full moon, etc., they only need gentle reminders or a quick self-regulation freeze game to come back to what is expected."

"The majority of students are able to regulate themselves better socially and emotionally….They are able to sustain elaborate play scenarios with multiple characters for extended periods of time! They are able to wait for their next turn. The students' growth in self-regulation and their excitement for learning [was the greatest reward of this year]!"

"Children are more independent and regulated. Ones who are not as regulated are regulated by others."

"A TOC [teacher-on-call, i.e., substitute teacher] recently commented how calm my class is."

Comments by *Tools* Teachers on the consequences of this:

"[Because] students are better regulated for sure than in past years, time is freed up for me to work with small groups. I have the freedom to work with small groups and help children learn at their own level; it helps provide students help where they need it and move them further faster. It is definitely more individualized and fits with our new curriculum. Students easily work in small groups and can self-regulate while I work with students who need support."

"They are very self-regulated so I am able to work with a small group without being distracted. This is a wonderful gift."

"The ability of my students to regulate their behaviour and to help those who still require some assistance has allowed me to be able to work with small groups as well as individually with specific students who require additional

assistance. I have never been able to effectively do this ever with kindergarten students before."

"Children are able to concentrate and be involved in activities for extended periods of time. That has made it far easier for me to work with individual students or small groups."

"The class (because of the students' self-regulation abilities) runs smoothly and seamlessly."

"Students are more independent and easier to manage….Management is easier and less stressful."

"At this time in the year [May]…there are many days where the children's energy is 'scattered' and they seem to march to the beat of their own drum."

"In my 3 years of teaching Kindergarten, I have never been able to effectively run small guided reading groups while the other students were engaged & working independently at literacy centres; this year I have been able to!"

Control-group teachers

"What I have enjoyed most is the growth I've seen. They were a MESS at the beginning. I felt like crying every afternoon. So many of them had low basic skills and very little independence, and their was a lot of fighting, crying, and meltdowns. They have come SUCH a long way. When I have been sick or facilitating PALS, every single TOC has said they were a joy to teach, which says to me that they are regulating well when I'm not there. – What has been most challenging has been sustaining my energy through the day. I feel that I need to verbalize much more with this group in terms of modeling appropriate behavior and social interactions. I feel that I need to hydrate and fuel with nutrition similar to the way I do when I am preparing for a long run."

"With the money [the $1,000 we gave each participating teacher for school supplies] I was able to purchase materials to create a softer, more natural atmosphere that would promote calm and would be conducive to self-regulation. Everyone remarks on what a calm, natural, and soothing environment I have in my classroom and I couldn't have made it that way without the funding."

"I've loved watching the children develop both socially and academically. At the beginning of the year, they could barely even sit on the carpet. They are so much better now."

"The classroom is quite diverse. There are… 4 children who have great difficulty self-regulating and controlling their actions/ impulses and or behaviour."

Comments on Children's JOY IN LEARNING and ENJOYMENT OF SCHOOL

Tools teachers

"I have enjoyed seeing the students get so excited about coming to school and learning about the topics/themes we had. They loved all the activities we did so much that many students refused to miss school even if they were sick."

"My greatest reward this year: Seeing the excitement of the students ready to learn and loving coming to school!"

"What I have you liked/ enjoyed most about my class this year is: All the learning that happened through play and dramatization. The smiles and joy. Hearing, "this is the best day, ever!" over and over again. The content parents who are extremely happy with what their children are doing."

"What I liked most about teaching this year: Students' enthusiasm towards learning and their pride in their development."

"What I liked most about teaching this year: The students excitement towards learning."

"The students' growth in self-regulation and their excitement for learning [was the greatest reward of this year]!"

"The students are more excited about learning and more engaged."

"Parents <u>love</u> it! They notice the student's excitement to learn and come to school!"

"The kids have fully bought in. There's no struggle in getting their attention or interest."

"Students are very motivated by subject matter, level of challenge and fun."

Control-group teachers

"I've loved watching the children develop both socially and academically."

"I enjoy my young students excitement and enthusiasm. And they learn so much in such a short time!"

Comments on FEELINGS ABOUT TEACHING

Tools teachers

"The fun and energy came back into my classroom and my teaching."

"I have seen so much success in my students' learning that I can't wait to begin teaching again next year now that I have a better understanding of the program and all of its benefits!"

"Learning all the new materials was worth all the effort and it will get easier every year."

"I am excited to have this year under my belt and to really be able to run with it next year. Learning was more exciting for me and the kids!"

Control-group teachers
