# Supplementary material for: Randomized control trial of Tools of the Mind: Marked benefits to kindergarten children and their teachers
Source: PLoS One. 2019 Sep 17;14(9):e0222447. doi: 10.1371/journal.pone.0222447 (PMC6748407; doi:10.1371/journal.pone.0222447)
Supplement: S6 File — (PDF) [file pone.0222447.s006.pdf]

Two Writing Samples from Kindergarten Children in *Tools of the Mind*

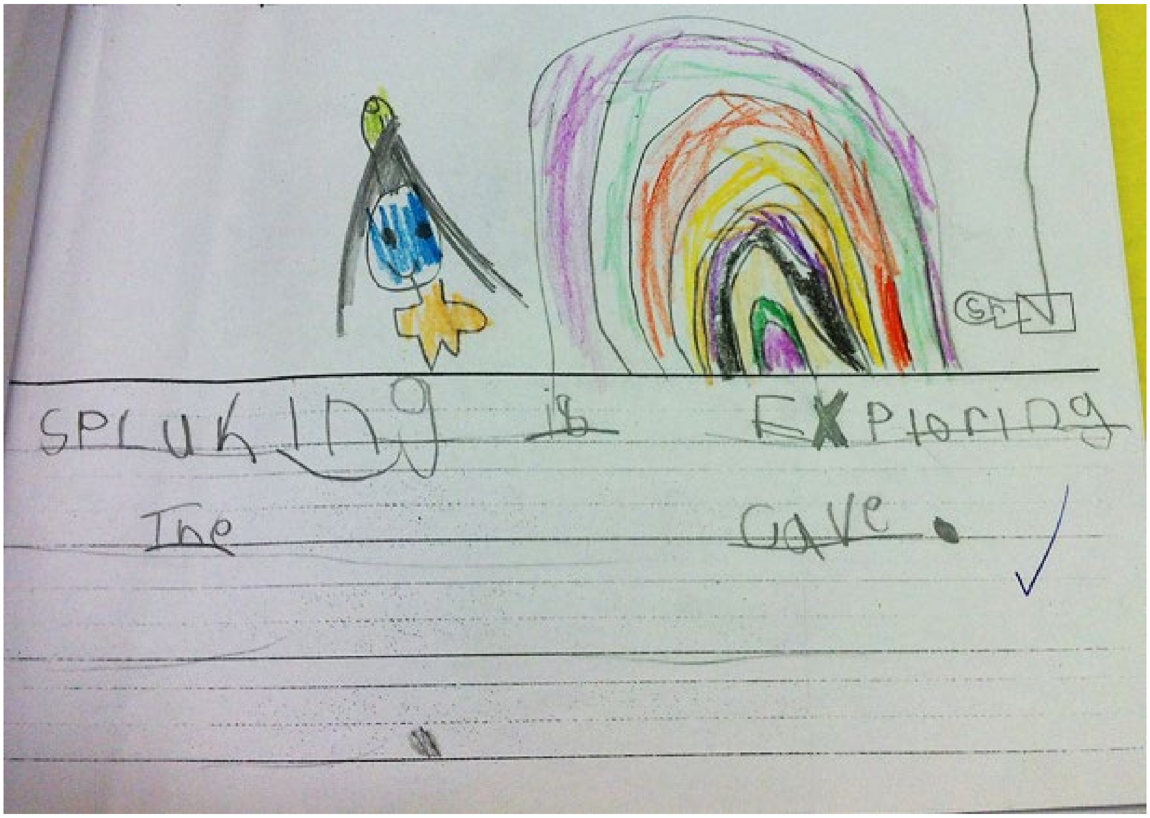

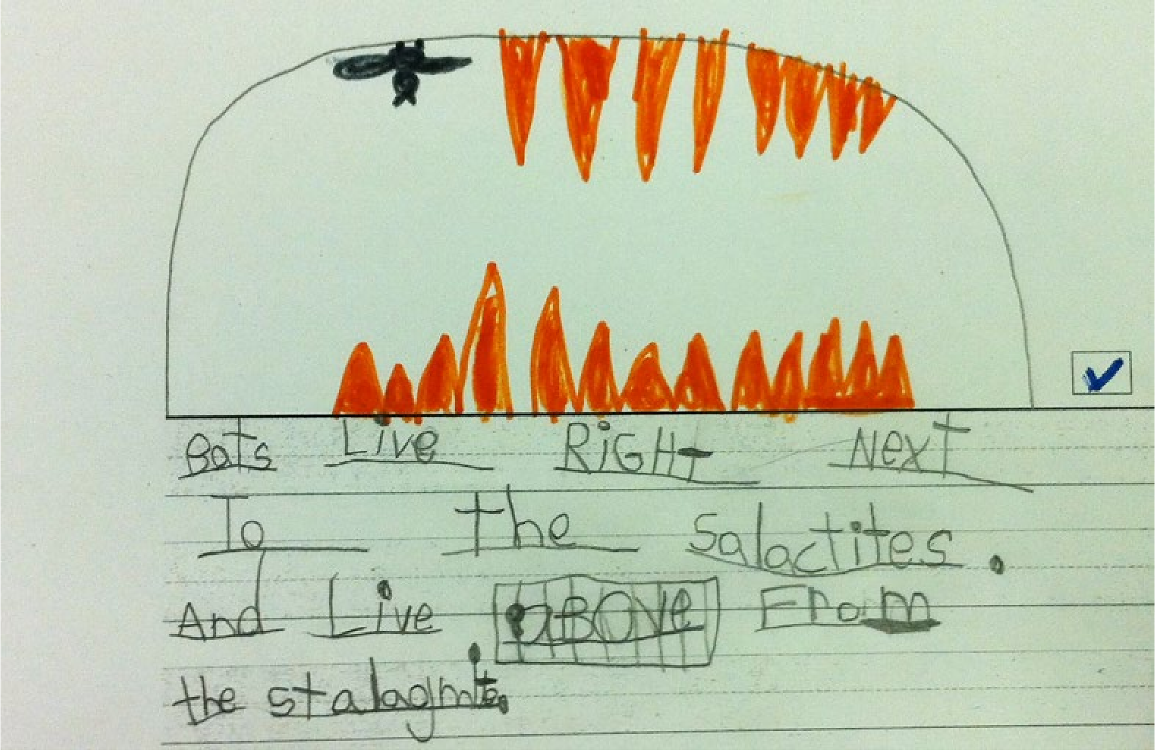

These were written the week after the children had had a lesson on caves.
